# Synthesis of 6- or 8-Carboxamido Derivatives of Imidazo[1,2-*a*]pyridines via a Heterogeneous Catalytic Aminocarbonylation Reaction

**DOI:** 10.3390/molecules29215048

**Published:** 2024-10-25

**Authors:** Enikő Nagy, Attila Máriás, Margit Kovács, Rita Skoda-Földes

**Affiliations:** 1 Research Group of Organic Synthesis and Catalysis, University of Pannonia, Egyetem u. 10, 8200 Veszprém, Hungary; nagyencsi6@gmail.com (E.N.); marias.attila2000@gmail.com (A.M.); 2 NMR Laboratory, University of Pannonia, Egyetem u. 10, 8200 Veszprém, Hungary; kovacs.margit@mk.uni-pannon.hu

**Keywords:** supported ionic liquid, palladium catalyst, amide, α-ketoamide

## Abstract

Imidazo[1,2-*a*]pyridines and especially their amide derivatives exhibit a wide range of favourable pharmacological properties. In this work, Pd-catalysed carbonylation was used for the first time for the introduction of the carboxamide moiety into positions 6 or 8. A recyclable Pd catalyst, with palladium immobilised on a supported ionic liquid phase decorated with pyridinium ions, was used efficiently for the conversion of 6- or 8-iodo derivatives to the products. In the case of 6-iodo derivatives, a competing mono- and double carbonylation could be observed in the reactions of aliphatic amines as nucleophiles, but under the proper choice of reaction conditions, good-to-excellent selectivities could be achieved towards either the corresponding amides or α-ketomides. The heterogeneous catalyst showed excellent recyclability and low Pd-leaching.

## 1. Introduction

In the past few years, the imidazo[1,2-*a*]pyridine scaffold has gained special attention due to the broad spectrum of biological activities exhibited by its derivatives [1], such as anti-tubercular [2], anti-cancer [3], antiviral [4], antibacterial [5], antiulcer [6], anti-HIV [7], anticonvulsant [8], analgesic and anti-inflammatory [9] effects. As a consequence, a great variety of synthetic methodologies towards these compounds was published [10,11], including metal-catalysed [12,13] or metal-free reactions [14], and photo- [15] or electrocatalytic protocols. With these strategies, imidazo[1,2-*a*]pyridines of diverse structures can be obtained, starting mainly with 2-aminopyridine, in which both the amino group and aromatic nitrogen have a basic/nucleophilic character enabling the attack of suitable electrophiles to form fused imidazoles. Other synthetic methodologies using pyridines, pyridinium salts or imidazole derivatives were also reported [16]. There are much fewer examples for the introduction of substituents into the preformed imidazo[1,2-*a*]pyridine core. This can be achieved either by CH activation in metal-catalysed [17] or photocatalytic [15] transformations, or by Pd-catalysed couplings of halide derivatives [18].

**Figure 1:**
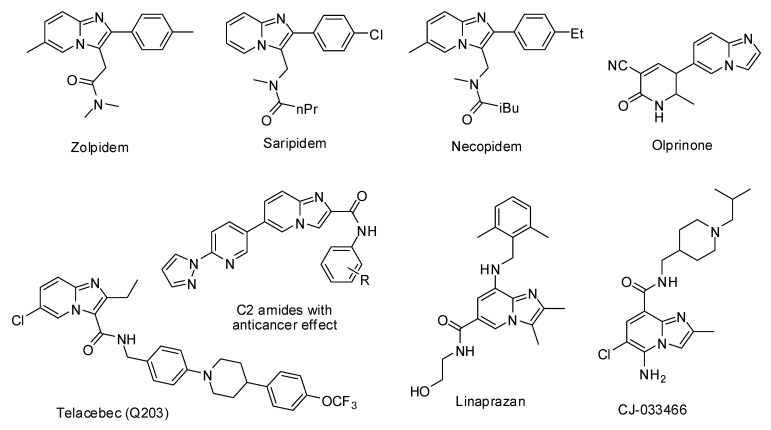
Marketed drugs and potential drug candidates incorporating the imidazo[1,2-*a*]pyridine core and an amide functionality.

Among marketed drugs and potential drug candidates based on the imidazo[1,2-*a*]pyridine core, there are several examples of amide derivatives. Commercialised imidazo[1,2-*a*]pyridine drugs with amide functionalities in the side chains include C3 substituted GABA receptor agonist Zolpidem [19] (Figure 1), used in the treatment of insomnia, and anxiolytic agents Necopide and Saripidem [20], as well as C8-functionalised phosphodiesterase inhibitor Olprinone [21], prescribed for treating heart failure.

Some carboxamides with the amide group attached directly to the imidazo[1,2-*a*]pyridine core also have promising biological effects. C3 amides (e.g., Telacebec (Q203)) exhibited antimycobacterial activity [22], and some C2 derivatives were found to be cytotoxic against different cancer cell lines (MCF-7, MDA-MB-231, A549 and DU-145) [23]. Linaprazan bearing the amide functionality in position 6, is a gastric hydrogen potassium pump inhibitor that can be used to control the secretion of gastric acid into the stomach [24]. An 8-carboxamide compound (CJ-033466), a selective, orally active, partial agonist for 5-HT_4_ receptors, displayed a stimulatory effect on gastric antral motility and produced a significant gastroprokinetic effect that is 30 times more potent than that of cisapride [25]. Similar derivatives were tested for 5-HT_2A_ serotonin receptor mediated disorders [26]. These amides were obtained by the introduction of an ester functionality to the C2, C3 [26] or C8 position [27] during the construction of the imidazo[1,2-*a*]pyridine core, followed by saponification and amidation. Linaprazan can be produced from a substituted nicotinamide derivative [24]. Das and colleagues showed that C2 [28] and C3 [29] derivatives could be prepared from the corresponding iodides in the presence of Pd(OAc)_2_ and bidentate ligands (dppe (1,2-bis(diphenylphosphino)ethane) and dppf (1,1′-bis(diphenylphosphino)ferrocene), respectively) using the CHCl_3_/KOH system as CO surrogate.

At the same time, to the best of our knowledge, the only example of an aminocarbonylation reaction carried out on the pyridine ring of imidazo[1,2-*a*]pyridines is the introduction of a *N*,*N*-dimethylamido functionality to C6 by the methodology mentioned above [28]. Moreover, under the proper conditions, Pd-catalysed aminocarbonylation can lead not only to amides, but also to ketocarboxamides [30]. The ketoamide motif is an important structural element of different natural products as well as drug candidates with sedative/hypnotic, anxiolytic, antitumor, antibacterial, antiviral and antiprion potency [31]. In the case of imidazo[1,2-*a*]pyridines, there is only one example of the synthesis of the latter type of compounds but with an alternative pathway; C3-substituted compounds were obtained via a regioselective copper-catalysed CH functionalisation with *N*,*N*-dialkyl acetamides in the presence of molecular oxygen as the oxidant [32].

As a continuation of our work concerning the application of recyclable SILP-Pd catalysts (SILP: Supported Ionic Liquid Phase) for the functionalisation of different skeletons [33,34,35,36], the present paper describes the preparation of 6- or 8-carboxamido-imidazo[1,2-*a*]pyridines, as well as 6-oxoamides, via aminocarbonylation using an immobilised palladium catalyst. To the best of our knowledge, there is no precedent for the application of a heterogeneous catalyst for the palladium-catalysed conversion of halogenoimidazo[1,2-*a*]pyridines. Our previous work [35] has proved that the recyclability of the catalyst and palladium leaching depend not only on the reaction conditions but also on the nature of the substrate. From this point of view, it is important to investigate the performance of a heterogeneous catalyst for the conversion of scaffolds different from simple iodoarenes, especially of those offering coordination sites for palladium that might lead to an increase in the loss of palladium.

## 2. Results and Discussion

**Scheme 1:**
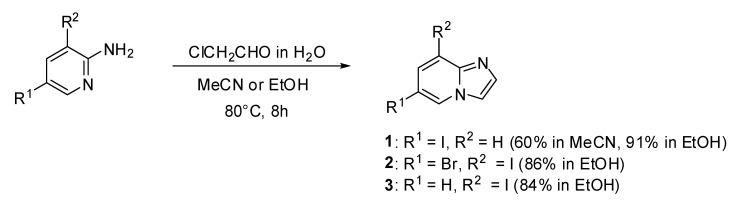
Synthesis of iodoimidazo[1,2-*a*]pyridine derivatives **1**–**3**.

The iodoimidazo[1,2-*a*]pyridine substrates **1**–**3** were obtained by the condensation of the corresponding iodo-2-aminopyridines with chloroacetaldehyde (Scheme 1) [37]. The original conditions in acetonitrile led to product **1** in moderate yield that could be increased considerably by replacing the solvent with ethanol. Under these conditions, the desired iodoimidazo[1,2-*a*]pyridines were obtained in 84–91% yield.

**Figure 2:**
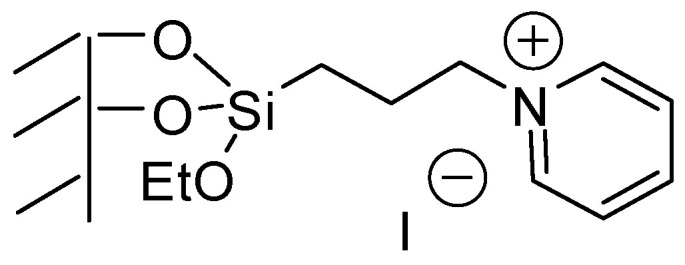
The structure of pyridinium SILP support used for the immobilisation of palladium.

Aminocarbonylation reactions were carried out with a heterogeneous catalyst obtained by the deposition of palladium on a SILP phase containing grafted pyridinium ions (Figure 2). Previously, this catalyst was found to be superior compared to those produced from imidazolium [38] or phosphonium SILPs [34] in the aminocarbonylation of iodobenzene and could also be applied efficiently in coupling reactions [36]. A detailed characterisation of the catalyst has been reported before [35].

Our previous experiences showed that CO pressure and temperature, as well as the choice of solvent and base, have a decisive effect on the outcome of the aminocarbonylation. These parameters were mainly selected based on our earlier findings, but some sets of conditions were tested in the carbonylation of 6-iodoimidazo[1,2-*a*]pyridine (**1**) and morpholine (**4a**), too (Table 1). In all of these reactions, the formation of both the amide (**5a**) and the α-ketoamide (**6a**) products was observed. In DMF, some side products (**7**, **8**), obtained via the decomposition of the solvent in the presence of the Pd catalyst [35,39], could also be detected in the reaction mixture. α-Ketoamide (**6a**) was produced with excellent selectivity at 30 bar CO pressure in DMF with Et_3_N as the base (entry 1). Although an almost complete conversion could be achieved even after 3 h of heating (entry 2), a longer reaction time was used in all other cases. This had been found to be favourable considering the efficient catalyst recycling; heating the reaction mixture for a longer time after the complete conversion of the substrate facilitates the re-precipitation of any leached metal on the surface of the support [33]. The application of other suitable solvent/base pairs [35] led to a lower **6a**/**5a** ratio (entries 3,4), although α-ketoamide (**6a**) remained the main product.

**Figure 3:**
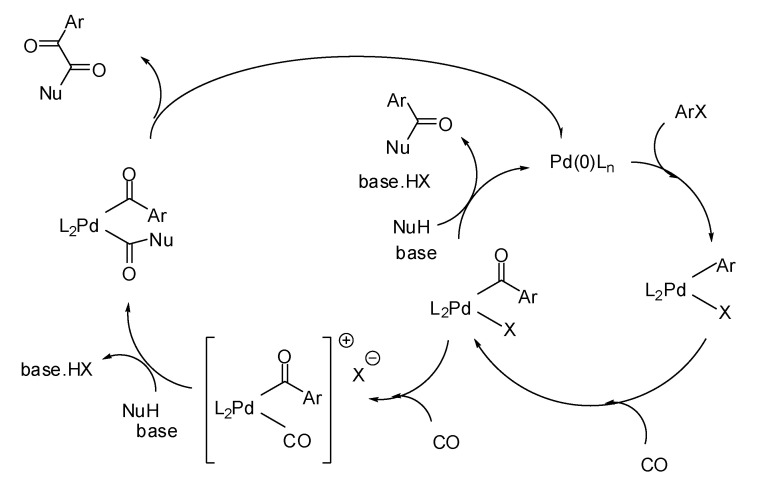
General mechanism of mono- and double carbonylation of aryl halides in the presence of nucleophiles.

In DMF, even a decrease in the pressure and the application of a higher temperature did not lead to amide **5a** as the main product, although the ratio of the latter increased compared to high-pressure experiments (entry 5). The preferential formation of α-ketoamide in polar solvents might be due to the supposed ionic intermediates in double carbonylation reactions, in contrast to the monocarbonylation route (Figure 3) [40]. In apolar solvents, amide **5a** (entries 6–8) could be produced in acceptable yield, although a considerable amount of α-ketoamide (**6a**) was also obtained in each reaction. Excellent selectivity towards amide **5a** was observed when carbonylation was carried out under 1 bar CO, but the reaction was much slower (entry 9).

**Figure 4:**
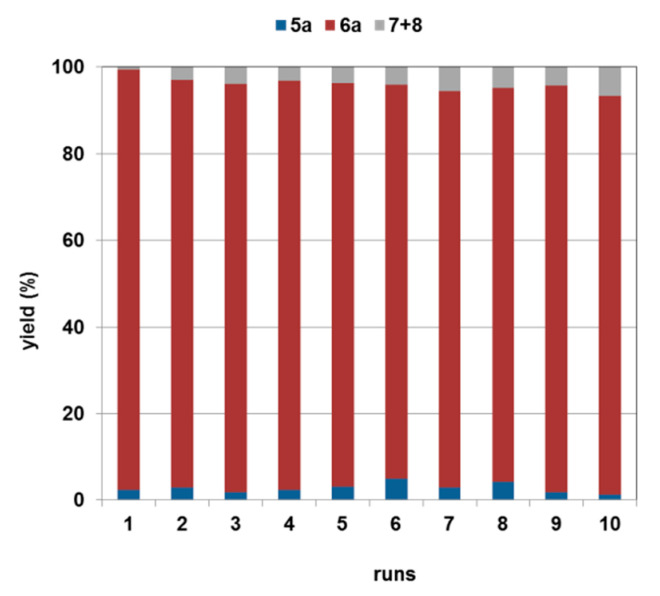
Recycling experiments in aminocarbonylation of 6-iodoimidazo[1,2-*a*]pyridine (**1**) and morpholine (**4a**) (Reaction conditions: 0.2 mmol **1**, 0.5 mmol **4a**, 0.25 mmol Et_3_N, catalyst **SILP-Pd** (with 2.8 μmol Pd-content), 2 mL DMF, 100 °C, 30 bar, 7 h).

**Figure 5:**
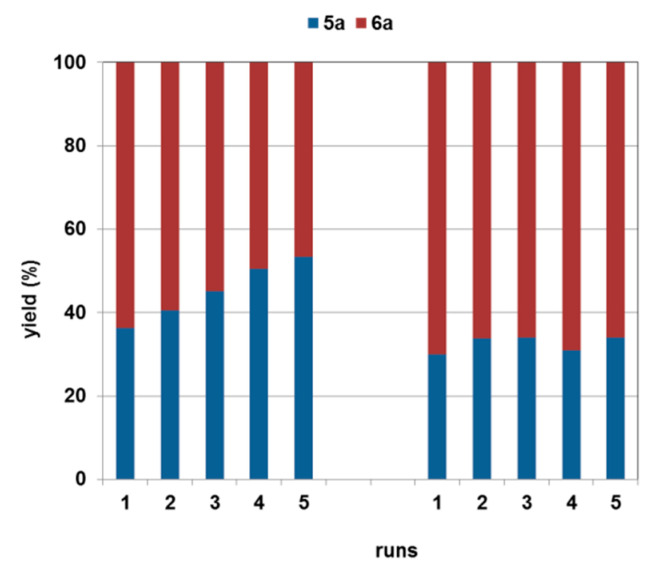
Recycling experiments in aminocarbonylation of 6-iodoimidazo[1,2-*a*]pyridine (**1**) and morpholine (**4a**) (Reaction conditions: 0.2 mmol **1**, 0.5 mmol **4a**, 0.25 mmol DBU, catalyst **SILP-Pd** (with 2.8 μmol Pd-content), 2 mL toluene (left) or 1,4-dioxane (right), 100 °C, 30 bar, 7 h).

**Figure 6:**
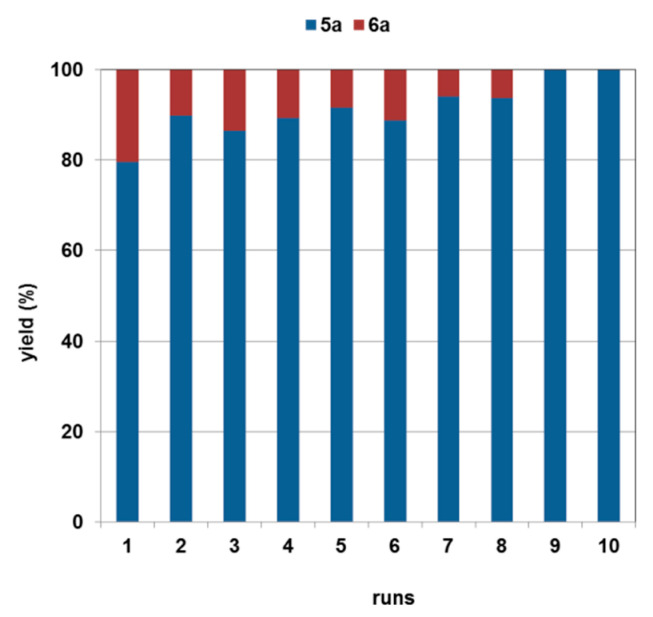
Recycling experiments in aminocarbonylation of 6-iodoimidazo[1,2-*a*]pyridine (**1**) and morpholine (**4a**) (Reaction conditions: 0.2 mmol **1**, 0.5 mmol **4a**, 0.25 mmol DBU, catalyst **SILP-Pd** (with 2.8 μmol Pd-content), 2 mL toluene, 120 °C, 5 bar, 7 h).

The recyclability of the catalyst and Pd-leaching were investigated under different conditions. At high pressure, good activity and excellent selectivity for α-ketoamide (**6a**) could be achieved in ten subsequent experiments in DMF (Figure 4). Only a slight increase in the percentage of side products **7** and **8** could be perceived. In toluene, a progressive rise in the ratio of the amide **5a** was observed in the recycling experiments, while the application of 1,4-dioxane did not lead to a noticeable change in selectivity on catalyst reuse (Figure 5). Under lower pressure and at a higher temperature, monocarbonylation prevailed in toluene (Figure 6). The ratio of the amide product **5a** was even enhanced when the catalyst was recycled, similarly to the high-pressure experiments carried out in this solvent. After the 8th cycle, exclusive formation of compound **5a** could be detected.

The palladium content of the reaction mixtures was determined by ICP-OES (Inductively Coupled Plasma Optical Emission Spectrometry) measurements (Table 2). A gradually increasing loss of palladium was detected in the recycling experiments in polar DMF (entries 1–3), while a reverse tendency (entries 6–10), or low Pd-content (entries 4, 5), could be observed in toluene or 1,4-dioxane. On comparing the experiments carried out under different conditions (entries 4, 5 and 8–10) in toluene, it can be concluded that the use of higher pressure hinders palladium leaching.

Based on the above findings, the aminocarbonylation of other aliphatic amines was carried out under two different sets of conditions, corresponding to those described in Table 1, entries 1 and 8, leading to amide (**5**) and ketoamide (**6**) derivatives as the main products, respectively (Table 3). Cyclic amines (**4b, c**) gave similar results to those obtained with morpholine (**4a**) (entries 3–6). A slightly lower selectivity was obtained at a high pressure with acyclic derivatives **4d** and **4e** (entries 7, 10), and their reactivity was evidently lower in experiments carried out with 5 bar CO (entries 8, 9 and 11, 12). The basicity of these amines are very similar to cyclic compounds **4b,c** so the differences in selectivity and reactivity can probably be explained by some steric hindrance of the alkyl chains in the case of these substrates.

The effect of different reaction conditions was also tested with an aromatic reaction partner (**4f**) (Table 4). Due to the lower nucleophilicity of aniline, lower conversion could be achieved compared to carbonylations with morpholine. However, the main difference lies in the high selectivity towards the amide product **5f** under all reaction conditions. This observation is in accordance with the previous findings of ourselves [38] and others [41,42]. The formation of ketoamide **6f** could be detected only at high pressures (entries 1–3), and even the use of 50 bar CO led to this product only in 8% of cases (entry 3). (Unfortunately, it could not be separated completely from amide **5f,** so it was characterised only by GC-MS.) It should also be mentioned that such a high pressure led to some decomposition of the catalyst and the deposition of Pd black inside the autoclave. At the same time, no such phenomenon was observed at 30 bar or below. Some increase in the ratio of side product **7** was noticed at lower pressure in DMF (entries 4,5). (Ketoamide **8** could also be detected in traces even under these conditions). In toluene, the complete conversion of substrate **1** to amide **5f** was achieved (entry 6). In spite of this, DMF was used as solvent for the preparation of amides of aniline derivatives **5g**–**i** (Table 5) due to the low solubility of these products in toluene, which hindered the separation of the product and the catalyst.

**Figure 7:**
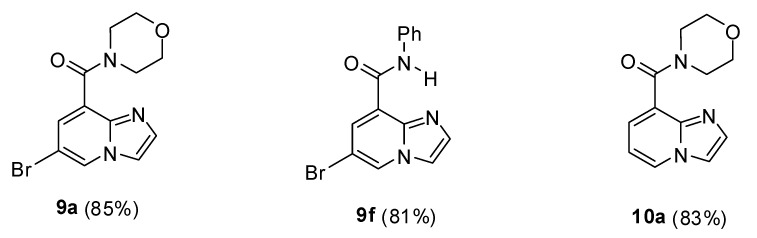
Products obtained by the aminocarbonylation of 8-iodoimidazo[1,2-*a*]pyridines **2** and **3**. (Reaction conditions: 0.2 mmol **2** or **3**, 0.5 mmol amine (**4a** or **4f**), 0.25 mmol Et_3_N, catalyst **SILP-Pd** (with 2.8 μmol Pd-content), 2 mL DMF, 100 °C, 30 bar, 7 h). In parenthesis: yields after column chromatography.

**Figure 8:**
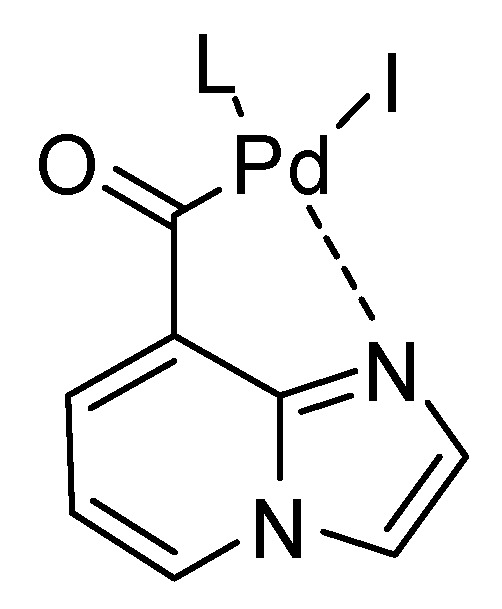
Assumed coordination of Pd to N(1) of 8-iodoimidazo[1,2-*a*]pyridine (**3**) in the acyl complex formed during carbonylation.

Interestingly, aminocarbonylation of the 8-iodo derivative **2** led to the exclusive formation of amides **9a** and **9f** (Figure 7), even at 30 bar CO pressure. Based on the results obtained with substrate **1**, the lack of the double carbonylation product is surprising in the reaction of the aliphatic amine **4a**. The formation of amide derivatives as the only products had been observed in the presence of SILP catalysts before, when very reactive iodoaromatics with strong electron withdrawing groups (e.g., NO_2_) had been used as substrates [34,35]. At the same time, a bromo substituent had had no such effect [34]. In order to shed some light on the influence of the structure of the substrate on the outcome of the reaction, an 8-iodoimidazo[1,2-*a*]pyridine (**3**) was also aminocarbonylated in the presence of morpholine (**4a**). Here again, only amide **10a** could be detected as the sole product of the reaction. In the case of Pd-catalysed amination reactions, comparable or only slightly improved results were obtained in position 8 relative to position 6 by Gueiffier et al. [43]. Decreased selectivity for double carbonylation was observed by us, starting from 1-iodonaphthalene, due to some steric hindrance, but still, the corresponding ketoamide was the main product [38]. Based on these observations, it is probable that the coordination of nitrogen in position 1 to Pd (Figure 8) hinders the entrance of the second CO ligand in the present case. It should also be mentioned that the bromo substituent remained intact under the present conditions.

## 3. Materials and Methods

### 3.1. General Information

Gas chromatographic and GC-MS measurements were carried out on a Hewlett Packard 5890 device equipped with an SPB-1 column and on a Hewlett Packard 5971A GC-MSD instruments using a DB-5MS column, respectively. ^1^H and ^13^C{^1^H} NMR spectra were acquired at 400.13 MHz and 100.62 MHz, respectively, on a Bruker Avance 400 spectrometer (Billerica, MA, USA) in CDCl_3_ or DMSO-d_6_. KBr pellets of samples were prepared to take FT-IR spectra on a Shimadzu IRAffinity-1S FT-IR spectrometer (Kyoto, Japan). The Pd content of reaction mixtures was determined using an ICP-OES Perkin Elmer Avio 550 Max instrument (Shelton, CT, USA). The preparation of the heterogeneous palladium catalyst was described before [35]. All other reactants were purchased from commercial sources and were used as received. Fine chemicals and solvents were purchased from Merck, Budapest, Hungary and VWR, Debrecen, Hungary, respectively, 

### 3.2. Synthetic Procedures

#### 3.2.1. Synthesis of Iodoimidazo[1,2-*a*]pyridine Derivatives **1**–**3** (Based on Ref. [37])

A mixture of a 2-amino-iodopyridine **1**, **2** or **3** (0.5 mmol) and chloroacetoaldehyde (50 m/m% solution in water, 114 µL, 0.9 mmol) was stirred in 11 mL acetonitrile at 80 °C for 8 h. The progress of the reaction was followed by TLC and gas chromatography. The solvent was removed in vacuo. The residue was dissolved in CH_2_Cl_2_ (15 mL) and was extracted with a saturated Na_2_CO_3_ solution (2 × 10 mL). The organic phase was dried on Na_2_SO_4_ and then the solvent was removed in vacuo. The products were isolated using column chromatography (silica, eluent: ethyl acetate/methanol = 50/1).

6-Iodoimidazo[1,2-*a*]pyridine (**1**) [37]: Yield: 91%, pale yellow solid, mp.: 127–130 °C, R_f_: 0.42 (EtOAc/MeOH = 50/1). ^1^H NMR (400.13 MHz, CDCl_3_) δ: 8.40 (dd, *J* = 1.6 Hz, 0.9 Hz, 1H); 7.58 (d, *J* = 0.8 Hz, 1H); 7.52–7.53 (m, 1H); 7.42 (dd, *J* = 9.4 Hz, 0.8 Hz, 1H); 7.32 (dd, *J* = 9.4 Hz, 1.6 Hz, 1H). ^13^C{^1^H} NMR (100.6 MHz, CDCl_3_) δ: 144.2; 134.1; 132.5; 130.8; 119.1; 112.3; 75.4. MS *m*/*z* (rel. int. %): 244 [M]^+^ (38); 127 (100,); 117 (31); 90 (33); 76 (9); 63 (8); 52 (38); 38 (48). IR: (cm^−1^) 713; 721; 799; 829; 874; 1130; 1306; 1337; 1417; 1501; 3063; 3105; 3125.

6-Bromo-8-iodoimidazo[1,2-*a*]pyridine (**2**): Yield: 86%, light brown solid, mp.: 145–147 °C, R_f_: 0.80 (EtOAc/MeOH = 50/1). ^1^H NMR (400.13 MHz, CDCl_3_) δ: 8.27 (d, *J* = 1.7 Hz, 1H); 7.74 (d, *J* = 1.7 Hz, 1H); 7.68–7.69 (m, 2H). ^13^C{^1^H} NMR (100.6 MHz, CDCl_3_) δ: 143.8; 136.5; 134.8; 126.2; 114.9; 106.8; 85.0. MS *m*/*z* (rel. int. %): 322/324 [M]^+^ (69); 195/197 (14); 168/170 (17); 143 (15); 127 (84); 116 (53); 89 (39); 62 (100); 52 (31); 38 (53). IR: (cm^−1^) IR: (cm^−1^) 718; 818; 860; 903; 1070; 1146; 1202; 1296; 1319; 1481; 1499; 3065; 3102.

8-Iodoimidazo[1,2-*a*]pyridine (**3**): Yield: 84%, pale green solid, mp.: 115–117 °C. R_f_: 0.56 (EtOAc/MeOH = 50/1). ^1^H NMR (400.13 MHz, CDCl_3_) δ 8.11 (dd, *J* = 6.9, 1.1 Hz, 1H); 7.70 (d, *J* = 1.0 Hz, 1H); 7.68 (d, *J* = 1.0 Hz, 1H); 7.65 (dd, *J* = 6.9 Hz, 1.1 Hz, 1H); 6.53 (t, *J* = 6.9 Hz, 1H). ^13^C{^1^H} NMR (100.6 MHz, CDCl_3_) δ = 144.9; 133.9; 126.1; 114.5; 113.3; 84.5. MS *m*/*z* (rel. int. %): 244 [M]^+^ (100); 127 (13); 117 (48); 90 (67); 63 (36); 62 (13); 39 (24) IR: (cm^−1^) 721; 738; 763; 771; 825; 894; 1138; 1204; 1300; 1494; 1618; 3026; 3092; 3129.

#### 3.2.2. General Procedure for Aminocarbonylation

In a typical experiment, the catalyst (containing 2.8 μmol Pd) was placed in a stainless-steel autoclave. The iodoimidazo[1,2-*a*]pyridine derivative (0.2 mmol), amine (0.5 mmol), base (0.25 mmol) and solvent (2 mL) were transferred into it under an inert atmosphere. The autoclave was charged with carbon monoxide (5 bar or 30 bar) and heated with stirring in an oil bath at 100 °C or 120 °C. After cooling to room temperature, the liquid phase was removed with a syringe. (In recycling experiments, the catalyst was reused.) The reaction mixture was analysed by gas chromatography. After the evaporation of the solvent, the products were purified by column chromatography (silica, eluent: ethyl acetate/ethanol 3/1 (**5a**, **5d**), ethyl acetate/ethanol 5/1 (**5c**, **6b**, **10**), ethyl acetate/ethanol 10/1 (**5e**), ethyl-acetate/methanol 3/1 (**5b**), ethyl-acetate/methanol 5/1 (**10a**), hexane/ethyl acetate 4/1 (**9f**), ethyl acetate/methanol/toluene 5/1/1 (**6a**, **6c**–**e**, **9a**), chloroform/ethyl acetate/methanol 10/2/1 (**5f**), dichloromethane/ethyl acetate/methanol 16/10/1 (**5g**), dichloromethane/ethyl acetate/methanol 8/5:2 (**5h**, **5i**)).

Preparation of the samples for the determination of Pd-content: After the removal of the solvent, the residue of the reaction mixture was dried in vacuum, weighed and 3 mL of cc. HNO_3_ was added. Then, the mixture was heated at 250 °C for 6 h. After cooling, it was transferred to a volumetric flask and was diluted to a total volume of 10 mL with deionised water.

Imidazo[1,2-*a*]pyridin-6-yl(morpholino)methanone (**5a**): yield: 75%, pale yellow solid, mp.:149–151 °C; R_f_: 0.55 (EtOAc/EtOH = 3/1). ^1^H NMR (400.13 MHz, CDCl_3_, 227K) δ: 8.42 (dd, *J* = 1.8 Hz, 1.0 Hz, 1H); 7.70 (d, *J* = 1.3 Hz, 1H); 7.67 (d, *J* = 1.3 Hz, 1H); 7.62–7.66 (m, 1H); 7.17 (dd, *J* = 9.3 Hz, 1.8 Hz, 1H); 3.78–3.82 (m, 4H); 3.65–3.71 (m, 2H); 3.54–3.59 (m, 2H). ^13^C{^1^H} NMR (100.6 MHz, CDCl_3_, 227K) δ:167.3; 144.6; 134.9, 126.9; 123.1, 120.6, 117.4, 113.6, 66.8, 66.7, 48.3; 42.7. MS *m*/*z* (rel. int. %): 231 [M]^+^ (39); 159 (12); 145 (100); 117 (76); 90 (52); 73 (48); 63 (47); 56 (100); 41 (39). IR: (cm^−1^) 744; 1024; 1113; 1222; 1286; 1311; 1425; 1438;1463; 1620; 1643; 2856; 2920; 2966; 3092.

Imidazo[1,2-*a*]pyridin-6-yl(pyrrolidin-1-yl)methanone (**5b**): yield: 82%, pale yellow solid, mp.: 94–96 °C, R_f_: 0.24 (EtOAc/MeOH = 3/1). ^1^H NMR (400.13 MHz, CDCl_3_) δ: 8.45 (dd, *J* = 1.6 Hz, 0.9 Hz, 1H); 7.67 (d, *J* = 1.2 Hz,1H); 7.59–7.62 (m, 2H); 7.32 (dd, *J* = 9.3 Hz, 1.6 Hz, 1H); 3.60–3.69 (m, 2H); 3.48–3.57 (m, 2H); 1.88–2.01 (m, 4H). ^13^C{^1^H} NMR (100.6 MHz, CDCl_3_, 227K) δ:166.5; 145.1; 134.9; 126.7; 123.5; 122.8; 117.3; 113.3; 49.8; 46.7, 26.7, 24.5. MS *m*/*z* (rel. int. %): [M]^+^ (15); 145 (56); 117 (59); 90 (29); 73 (5); 63 (26); 56 (10); 41 (100); 39 (55). IR: (cm^−1^) 715; 732; 1140; 1311; 1423; 1435; 1456; 1506; 1539; 1558; 1606; 1635; 1645; 2875; 2981; 3088; 3130.

Imidazo[1,2-*a*]pyridin-6-yl(piperidin-1-yl)methanone (**5c**): yield: 82%, brownish oil, R_f_: 0.26 (EtOAc/EtOH = 5/1). ^1^H NMR (400.13 MHz, CDCl_3_, 233K) δ: 8.35 (dd, *J* = 1.5 Hz, 1.0 Hz, 1H); 7.66 (d, *J* = 1.2 Hz, 1H); 7.63–7.64 (m, 1H); 7.61 (d, *J* = 9.2 Hz, 1H); 7.15 (dd, *J* = 9.2 Hz, 1.5 Hz, 1H); 3.63–3.72 (m, 2H); 3.38–3.46 (m, 2H); 1.61–1.71 (m, 4H); 1.49–1.58 (m, 2H). ^13^C{^1^H} NMR (100.6 MHz, CDCl_3_, 233K) δ: 167.1; 144.7; 134.6; 126.1; 123.3; 121.7; 117.2; 113.4; 49.0; 43.5; 26.5; 25.4; 24.3. MS *m*/*z* (rel. int. %): 229 [M]^+^ (7); 145 (39); 117 (28); 90 (14); 73 (2); 63 (18); 55 (59); 42 (100); 39 (48). IR: (cm^−1^) 744; 819; 1006; 1132; 1221; 1288; 1311; 1425; 1444; 1470; 1620; 1641; 2855; 2938; 3089.

*N*,*N*-diethylimidazo[1,2-*a*]pyridine-6-carboxamide (**5d**). yield: 76%, pale yellow solid, mp.: 55–59 °C, R_f_: 0.30 (EtOAc/EtOH = 5/1). ^1^H NMR (400.13 MHz, CDCl_3_, 227 K) δ: 8.7 (dd, *J* = 1.0 Hz, 1.6 Hz, 1H); 7.66–7.72 (m, 3H); 7.25 (dd, *J* = 1.6 Hz, 9.5Hz, 1H); 3.54 (q, *J* = 7.5 Hz, 2H); 3.35 (q, *J* = 7.5 Hz, 2H); 1.24 (t, *J* = 7.6 Hz, 3H); 1.20 (t, *J* = 7.6 Hz, 3H). ^13^C{^1^H} NMR (100.6 MHz, CDCl_3_, 227 K) δ: 167.7; 144.2; 133.7; 125.3; 123.8; 122.9; 117.1; 113.6; 43.6; 39.6; 14.5; 12.8. MS *m*/*z* (rel. int. %): 217 [M]^+^ (29); 146 (19); 145 (100); 118 (31); 117 (47); 90 (21); 72 (11); 63 (14); 42 (9); 39 (8). IR: (cm^−1^) 752; 795; 822; 1078; 1134; 1312; 1423; 1456; 1616; 1636; 2928; 2967; 3032; 3099.

*N*,*N*-dibutylimidazo[1,2-*a*]pyridine-6-carboxamide (**5e**): yield: 82%, pale brown oil, R_f_: 0.34 (EtOAc/EtOH = 10/1). ^1^H-NMR (400.13 MHz, CDCl_3_, 227 K) δ: 8.28–8.32 (m, 1H); 7.61–7.70 (m, 3H); 7.11–7.18 (m, 1H); 3.43 (t, *J* = 7.5 Hz, 2H); 3.23 (t, *J* = 7.5 Hz, 2H); 1.54– 1.66 (m, 2H); 1.45–1.54 (m, 2H); 1.34 (quart, *J* = 7.5 Hz, 2H); 1.12 (quart, *J* = 7.5 Hz, 2H); 0.93 (t, *J* = 7.5 Hz, 3H); 0.80 (t, *J* = 7.5 Hz, 3H). ^13^C{^1^H} NMR (100.6 MHz, CDCl_3_, 227 K) δ: 168.1; 144.4; 134.3; 125.3; 123.2; 122.6; 117.3; 113.4; 49.0; 44.7; 30.7; 29.3; 20.3; 19.8; 14.2; 13.9. MS *m*/*z* (rel. int. %): 273 [M]^+^ (13); 244 (6); 230 (6); 145 (100); 117 (30); 90 (11); 63 (6); 41 (9); 39 (5). IR: (cm^−1^) 669; 748; 816; 1217; 1312; 1458; 1618; 1638; 2931; 2949.

*N*-Phenylimidazo[1,2-*a*]pyridine-6-carboxamide (**5f**): yield: 84%, pale yellow solid, mp.: 218–219 °C, R_f_: 0.39 (CHCl_3_/EtOAc/MeOH = 10/2/1). ^1^H NMR (400.13 MHz, DMSO-d_6_) δ: 10.24 (s, 1H); 9.24–9.27 (m, 1H); 8.08–8.13 (m, 1H); 7.72–7.79 (m, 3H); 7.68–7.71 (m, 1H); 7.67 (d, *J* = 9.2 Hz, 1H); 7.34–7.40 (m, 2H); 7.09–7.15 (m, 1H). ^13^C{^1^H} NMR (100.6 MHz, DMSO-d_6_) δ: 163.4; 143.3; 138.9; 134.6; 128.9; 128.7; 123.8; 123.2; 120.3; 120.2; 116.2; 114.4. MS *m*/*z* (rel. int. %): 237 [M]^+^ (41); 145 (100); 117 (56); 90 (33); 77 (6); 63 (34); 52 (8); 39 (34). IR: (cm^−1^) 742; 813; 1132; 1203; 1238; 1313; 1442; 1494; 1558; 1670; 3032; 3138.

*N*-(4-Nitrophenyl)imidazo[1,2-*a*]pyridine-6-carboxamide (**5g**): yield: 78%, pale yellow solid, mp.: 276–278 °C, R_f_: 0.47 (CH_2_Cl_2_/EtOAc/MeOH = 16/10/1). ^1^H NMR (400.13 MHz, DMSO-d_6_) δ: 10.99 (s, 1H); 9.37–9.41 (m, 1H); 8.26–8.33 (m, 2H); 8.15–8.20 (m, 1H); 8.04–8.11 (m, 2H); 7.85 (dd, *J* = 9.4 Hz, 1.8 Hz, 1H); 7.77–7.80 (m, 1H); 7.74 (d, *J* = 9.4 Hz, 1H). ^13^C{^1^H} NMR (100.6 MHz, DMSO-d_6_) δ: 164.0; 145.2; 144.1; 142.6; 133.5; 129.7; 124.8; 124.2; 120.1; 119.9; 115.8; 114.7. MS *m*/*z* (rel. int. %): 282 [M]^+^ (4); 237 (26); 145 (69); 117 (48); 92 (26); 90 (45); 73 (26); 65 (100); 52 (33); 39 (56). IR: (cm^−1^) 750; 850; 1315; 1338; 1506; 1541; 1558; 1683; 3064; 3336.

*N*-(4-Methylphenyl)imidazo[1,2-*a*]pyridine-6-carboxamide (**5h**): yield: 67%, light brown solid, mp.: 208–210 °C, R_f_: 0.51 (CH_2_Cl_2_/EtOAc/MeOH = 8/5/2). ^1^H NMR (400.13 MHz, DMSO-d_6_) δ: 10.32 (s, 1H); 9.28 (dd, *J* = 1.9Hz, 0.9Hz, 1H); 8.12–8.14 (m, 1H); 7.81 (dd, *J* = 9.3Hz, 2.3Hz, 1H); 7.74 (d, 2.3 Hz, 1H); 7.70 (d, *J* = 9.3Hz, 1H); 7.63–7.67 (m, 2H); 7.16–7.19 (m, 2H); 2.29 (s, 3H). ^13^C{^1^H} NMR (100.6 MHz, DMSO-d_6_) δ: 162.8; 143.9; 136.1; 133.2; 132.6; 128.9; 128.7; 123.9; 120.5; 120.19; 115.4; 114.3; 20.3. MS *m*/*z* (rel. int. %): 251 [M]^+^ (26); 145 (92); 117 (87); 106 (62); 90 (59); 77 (100); 65 (52); 52 (37); 39 (37). IR: (cm^−1^) 750; 808; 1122; 1309; 1512; 1602; 1618; 1664; 2931; 3032; 3292.

*N*-(4-Methoxyphenyl)imidazo[1,2-*a*]pyridine-6-carboxamide (**5i**): yield: 64%, light brown solid, mp.: 210–213 °C, R_f_: 0.47 (CH_2_Cl_2_/EtOAc/MeOH = 16/10/1). ^1^H NMR (400.13 MHz, DMSO-d_6_) δ:10.28 (s, 1H); 9.27–9.29 (m, 1H); 8.13 (s, 1H); 7.81 (dd, *J* = 9.4 Hz, 1.7 Hz, 1H); 7.72–7.75 (m, 1H); 7.65–7.72 (m, 3H); 6.92–6.97 (m, 2H); 3.75 (s, 3H). ^13^C{^1^H} NMR (100.6 MHz, DMSO-d_6_) δ: 162.8; 155.7; 144.2; 133.6; 131.9; 128.8;123.9; 122.0; 120.6; 115.7; 114.5; 113.8; 55.2. MS *m*/*z* (rel. int. %): 267 [M]^+^ (19); 223 (16); 178 (5); 145 (24); 133 (12); 122 (33); 117 (21); 90 (16); 73 (100); 63 (13); 52 (25). IR: (cm^−1^) 829;1024; 1251; 1515; 1529; 1541; 1622; 1649; 2841; 2968; 3088; 3344.

1-(Imidazo[1,2-*a*]pyridin-6-yl)-2-morpholinoethane-1,2-dione (**6a**): yield: 91%, white solid, mp.: 103–106 °C, R_f_: 0.61 (EtOAc/toluene/MeOH = 5/1/1). ^1^H NMR (400.13 MHz, CDCl_3_) δ: 8.94–8.98 (m, 1H); 7.63–7.77 (m, 4H); 3.75–3.82 (m, 4H); 3.65–3.71 (m, 2H); 3.42–3.48 (m, 2H). ^13^C{^1^H} NMR (100.6 MHz, CDCl_3_) δ: 187.6; 164.2; 145.9; 135.9; 133.0; 122.6; 120.4; 118.2; 114.4; 66.9; 66.7; 46.7; 42.1. MS *m*/*z* (rel. int. %): 259 [M]^+^ (19); 145 (100); 117 (74); 90 (501); 70 (52); 63 (58); 56 (55); 42 (65); 39 (19). IR: (cm^−1^) 760; 921; 981; 1109; 1172; 1273; 1317; 1435; 1635; 1664; 2866; 2933; 3101; 3144.

1-(Imidazo[1,2-*a*]pyridin-6-yl)-2-(pyrrolidin-1-yl)ethane-1,2-dione (**6b**): yield: 84%, light brown solid, decomposition at 170 °C, R_f_: 0.43 (EtOAc/MeOH = 5/1). ^1^H NMR (400.13 MHz, CDCl_3_) δ: 9.17–9.21 (m, 1H); 7.81 (dd, *J* = 9.4Hz, 1.7Hz, 1H); 7.68–7.76 (m, 3H); 3.63–3.69 (m, 2H); 3.52–3.59 (m, 2H); 1.94–2.02 (m, 4H). ^13^C{^1^H} NMR (100.6 MHz, CDCl_3_) δ: 187.5; 163.1; 145.5; 134.9; 133.6; 123.7; 120.7; 117.7; 114.5; 47.4; 46.1; 26.2; 24.0. MS *m*/*z* (rel. int. %): 243 [M]^+^ (16); 145 (47); 117 (67); 98 (65); 90 (52); 70 (12); 63 (57); 55 (100); 41 (66); 39 (52). IR: (cm^−1^) 692; 748; 1124; 1167; 1294; 1315; 1431; 1456; 1506; 1521; 1628; 1683; 2879; 2974.

1-(Imidazo[1,2-*a*]pyridin-6-yl)-2-(piperidin-1-yl)ethane-1,2-dione (**6c**): yield: 79%, pale brown solid, mp.: 58–60 °C, R_f_: 0.61 (EtOAc/toluene/MeOH = 5/1/1). ^1^H NMR (400.13 MHz, CDCl_3_) δ: 8.90 (dd, *J* = 1.6Hz, 1.0Hz, 1H); 7.66–7.75 (m, 4H); 3.70–3.74 (m, 2H); 3.34–3.38 (m, 2H); 1.69–1.75 (m, 4H); 1.57–1.64 (m, 2H). ^13^C{^1^H} NMR (100.6 MHz, CDCl_3_) δ: 188.6; 164.3; 145.8; 135.8; 132.6; 122.6; 120.5; 118.0; 114.4; 47.4; 42.7; 26.4; 25.6; 24.4. MS *m*/*z* (rel. int. %): 257 [M]^+^ (8); 145 (46); 117 (89); 112 (54); 90 (52); 69 (66); 63 (47); 56 (37); 41 (100); 39 (53). IR: (cm^−1^) 758; 822; 852; 875; 914; 974; 1121; 1175; 1250; 1304; 1433; 1628; 1665; 2870; 2947; 3145.

*N*,*N*-Diethyl-2-(imidazo[1,2-*a*]pyridin-6-yl)-2-oxoacetamide (**6d**): yield: 75%, light brown solid, mp.: 89–91 °C, R_f_: 0.60 (EtOAc/toluene/MeOH = 10/2/3). ^1^H NMR (400.13 MHz, CDCl_3_) δ: 8.88 (dd, *J* = 1.7, 1.1 Hz, 1H); 7.66–7.73 (m, 4H); 3.56 (q, *J* = 7.2 Hz, 2H); 3.29 (q, *J* = 7.2 Hz, 2H); 1.29 (t, *J* = 7.2 Hz, 3H); 1.20 (t, *J* = 7.2 Hz, 3H). ^13^C{^1^H} NMR (100.6 MHz, CDCl_3_) δ: 188.4; 165.6; 146.0; 136.1; 132.6; 122.6; 120.4; 118.2; 114.3; 42.6; 39.5; 14.5; 13.0. MS *m*/*z* (rel. int. %): 245 [M]^+^ (23); 217 (19); 145 (73); 117 (45); 100 (99); 90 (27); 72 (100); 63 (26); 56 (4); 44 (32); 42 (10); 39 (11). IR: (cm^−1^) 738; 767; 849; 1020; 1113; 1261; 1311; 1431; 1624; 1633; 1658; 2937; 2976; 3155.

*N*,*N*-Dibutyl-2-(imidazo[1,2-*a*]pyridin-6-yl)-2-oxoacetamide (**6e**): yield: 71%, light brown solid, mp.: 71–72 °C, R_f_: 0.65 (EtOAc/toluene/MeOH = 5/1/1). ^1^H NMR (400.13 MHz, CDCl_3_) δ: 8.88 (dd, *J* = 1.4 Hz, 1.0 Hz, 1H); 7.66–7.75 (m, 4H, 2-H); 3.47–3.52 (m, 2H); 3.18– 3.24 (m, 2H); 1.62–1.70 (m, 2H); 1.52–1.60 (m, 2H); 1.41 (sext, *J* = 7.3 Hz, 2H); 1.21 (sext, *J* = 7.3 Hz, 2H); 0.99 (t, *J* = 7.3 Hz, 3H); 0.84 (t, *J* = 7.3 Hz, 3H). ^13^C{^1^H} NMR (100.6 MHz, CDCl_3_) δ: 188.3; 165.9; 145.9; 135.9; 132.5; 122.9; 120.6; 118.1; 114.3; 47.8; 44.7; 31.0; 29.6; 20.4; 20.0; 14.0; 13.8. MS *m*/*z* (rel. int. %): 301 [M]^+^ (8); 273 (9); 156 (30); 145 (41); 117 (28); 100 (21); 90 (13); 63 (9); 57 (100); 41 (35); 39 (7). IR: (cm^−1^) 679; 738; 871; 949; 1182; 1282; 1313; 1429; 1630; 1636; 1683; 2866; 2955; 3098.

*N*,*N*-Dimethylimidazo[1,2-*a*]pyridine-6-carboxamide (**7**): MS *m*/*z* (rel. int. %): 89 [M]+ (38); 145 (95); 117 (79); 90 (27); 73 (11); 63 (37); 52 (14); 42 (100); 39 (14).

2-(Imidazo[1,2-a]pyridin-6-yl)-N,N-dimethyl-2-oxoacetamide (**8**): MS *m*/*z* (rel. int. %): 217 [M]+ (8); 145 (27); 117 (29); 90 (15); 72 (100); 63 (20); 56 (18); 42 (60); 39 (6).

(6-Bromoimidazo[1,2-*a*]pyridin-8-yl)(morpholino)methanone (**9a**): yield: 85%, brown solid, mp.: 164–167 °C, R_f_: 0.51 (EtOAc/toluene/MeOH = 5/1/1).^1^H-NMR (400.13 MHz, CDCl_3_) δ: 8.34 (d, *J* = 1.6 Hz, 1H); 7.67 (d, *J* = 1.1 Hz, 1H); 7.60 (d, *J* = 1.1 Hz, 1H); 7.34 (d, *J* = 1.6 Hz, 1H); 3.86–3.92 (m, 2H); 3.80–3.85 (m, 2H); 3.63–3.70 (m, 2H); 3.29–3.37 (m, 2H). ^13^C{^1^H} NMR (100.6 MHz, CDCl_3_) δ: 164.3; 140.4; 135.1; 127.0; 126.8; 126.6; 113.3; 106.6; 67.0; 66.8; 47.8; 42.7. MS *m*/*z* (rel. int. %): 309/311 [M]^+^ (1); 195/197 (13); 117 (13); 89 (11); 76 (4); 62 (13); 56 (100); 54 (21); 42 (25); 39 (4). IR: (cm^−1^) 715; 825; 995; 1107; 1141; 1207; 1281; 1300; 1437; 1458; 1491; 1546; 1624; 2853; 2905; 3036.

6-Bromo-*N*-phenyl-imidazo[1,2-*a*]pyridine-8-carboxamide (**9f**): yield: 81%, pale yellow solid, mp.: 204–207 °C, R_f_: 0.47 (hexane/EtOAc = 4/1). ^1^H NMR (400.13 MHz, DMSO-d_6_) δ: 12.25 (s, 1H); 9.15–9.19 (m, 1H); 8.11–8.13 (m, 1H); 8.06–8.09 (m, 1H); 7.82 (s, 1H); 7.77 (d, *J* = 7.7 Hz, 2H); 7.42 (t, *J* = 7.7 Hz, 2H); 7.17 (t, *J* = 7.7 Hz, 1H). ^13^C{^1^H} NMR (100.6 MHz, DMSO-d_6_) δ: 159.3; 140.9; 137.9; 132.9; 130.4; 129.8; 128.9 (2C); 124.2; 120.7; 119.6 (2C); 114.6; 105.4. MS *m*/*z* (rel. int. %): 315/317 [M]^+^ (2); 195/197 (5); 168/170 (1); 143 (3); 117 (12); 91 (27); 77 (10); 73 (46); 65 (100); 51 (14); 39 (42). IR: (cm^−1^) 721; 752; 810; 870; 902; 923; 1022; 1076 1148; 1224; 1276; 1302; 1313; 1489; 1564; 1599; 1667; 3064; 3086; 3111; 3136.

Imidazo[1,2-*a*]pyridin-8-yl(morpholino)methanone (**10a**): yield: 83%, pale brown solid, mp.: 137–140 °C, R_f_: 0.27 (EtOAc MeOH = 5/1). ^1^H NMR (400.13 MHz, DMSO-d_6_) δ: 8.62 (dd, *J* = 6.8, 1.2 Hz, 1H); 8.02 (d, *J* = 1.2 Hz, 1H); 7.61 (d, *J* = 1.2 Hz, 1H); 7.25 (dd, *J* = 6.8 Hz, 1.2 Hz, 1H); 6.95 (t, *J* = 6.8 Hz, 1H); 3.67–3.70 (m, 4H); 3.52 (t, *J* = 4.3 Hz, 2H); 3.15 (t, *J* = 4.3 Hz, 2H). ^13^C{^1^H} NMR (100.6 MHz, DMSO-d_6_) δ: 165.0; 141.0; 133.5; 127.8; 125.2; 123.1; 113.8; 111.7; 66.3; 66.0; 47.11; 41.9. MS *m*/*z* (rel. int. %): 231 [M]^+^ (4); 145 (21); 118 (100); 90 (17); 63 (7); 56 (5); 42 (2) 39 (5). IR: (cm^−1^) 716; 752; 993; 1105; 1219; 1284; 1310; 1456; 1645; 2857; 2962; 3101; 3142.

## 4. Conclusions

Iodoimidazo[1,2-*a*]pyridines with either a 6-carboxamide or 6-ketoamide functionality were produced with excellent selectivity and good yield from 6-iodoimidazo[1,2-*a*]pyridine and aliphatic amines, using a heterogeneous palladium catalyst. In contrast, exclusive monocarbonylation took place during the conversion of 8-iodo derivatives that might be attributed to the coordination of palladium to N(1) nitrogen of the imidazopyridine ring. Also, the selective formation of amide products was observed in the presence of aromatic amines as nucleophiles, irrespective of the position of the iodo substituent of the imidazo[1,2-*a*]pyridine substrate. The catalyst presented good recyclability and a low loss of palladium under optimised reaction conditions, both for mono- and double carbonylation reactions. The procedures described resulted in the formation of twelve novel amide derivatives together with five new α-ketoamides.

## Figures and Tables

**Table 1:** Aminocarbonylation of 6-iodoimidazo[1,2-*a*]pyridine (**1**) in the presence of morpholine (**4a**) ^a^.

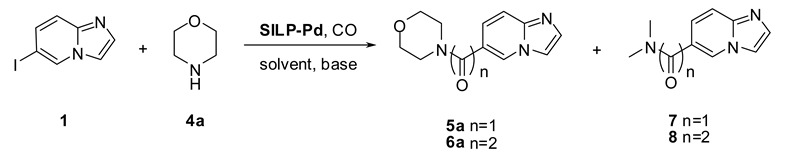								
Entry	Temp. [°C]	Pressure [bar]	Solvent	Base	Conv. [%] ^b^	Ratio of Products ^b^		
						5a [%]	6a [%]	7 + 8 [%]
1	100	30	DMF	Et_3_N	100	2	97	1
2 ^c^	100	30	DMF	Et_3_N	>99	4	95	1
3	100	30	1,4-dioxane	DBU	100	30	70	-
4	100	30	toluene	DBU	100	36	64	-
5	120	5	DMF	Et_3_N	100	34	61	5
6	120	5	1,4-dioxane	DBU	100	77	23	-
7	120	5	THF	DBU	100	70	30	-
8	120	5	toluene	DBU	100	82	18	-
9	120	1	toluene	DBU	45	92	8	-

^a^ Reaction conditions: 0.2 mmol **1**, 0.5 mmol **4a**, 0.25 mmol base, catalyst **SILP-Pd** (with 2.8 μmol Pd-content), 2 mL solvent, 7 h ^b^ Determined by GC. ^c^ Reaction time: 3 h.

**Table 2:** Pd-leaching in recycling experiments.

Entry	Temp. [°C]	Pressure [bar]	Solvent	Base	Run	Pd-Leaching [%] ^e^
1 ^a^	100	30	DMF	Et_3_N	1	1.7
2 ^a^	100	30	DMF	Et_3_N	2	2.4
3 ^a^	100	30	DMF	Et_3_N	5	4.4
4 ^b^	100	30	toluene	DBU	1	<0.5
5 ^b^	100	30	toluene	DBU	2	<0.5
6 ^c^	100	30	1,4-dioxane	DBU	1	3.2
7 ^c^	100	30	1,4-dioxane	DBU	2	0.8
8 ^d^	120	5	toluene	DBU	1	2.7
9 ^d^	120	5	toluene	DBU	2	0.8
10 ^d^	120	5	toluene	DBU	5	<0.5

^a^: See Figure 3. ^b^: See Figure 4, left. ^c^: See Figure 4, right. ^d^: See Figure 5. ^e^: Determined by ICP, % of original Pd-loading.

**Table 3:** Aminocarbonylation of 6-iodoimidazo[1,2-*a*]pyridine (**1**) in the presence of aliphatic amines (**4a**–**e**).

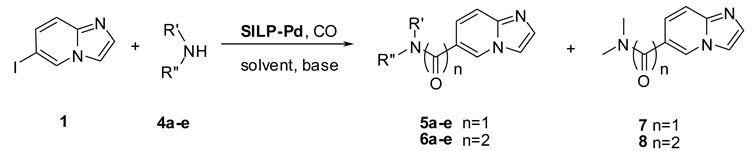									
Entry	Amine	R′	R″	Method ^a^	Conv. [%] ^b^	Ratio of Products ^b^			Yield [%] ^c^
						5 [%]	6 [%]	7 + 8 [%]	
1	**4a**	(CH_2_)_2_O(CH_2_)_2_		A	100	2	97	1	91 (**6a**)
2	**4a**	(CH_2_)_2_O(CH_2_)_2_		B	100	82	18	-	75 (**5a**)
3	**4b**	(CH_2_)_4_		A	99	8	91	1	84 (**6b**)
4	**4b**	(CH_2_)_4_		B	100	87	13	-	82 (**5b**)
5	**4c**	(CH_2_)_5_		A	98	3	97	-	80 (**6c**)
6	**4c**	(CH_2_)_5_		B	100	90	10	-	82 (**5c**)
7	**4d**	Et	Et	A	100	18	82	-	75 (**6d**)
8	**4d**	Et	Et	B	94	81	19	-	n.d.
9 ^d^	**4d**	Et	Et	B	100	83	17	-	76 (**5d**)
10	**4e**	Bu	Bu	A	100	21	79	-	71(**6e**)
11	**4e**	Bu	Bu	B	68	80	20	-	n.d.
12 ^e^	**4e**	Bu	Bu	B	100	88	12	-	82 (**5e**)

^a^ Reaction conditions: 0.2 mmol **1**, 0.5 mmol amine (**4**), 0.25 mmol base, catalyst **SILP-Pd** (with 2.8 μmol Pd-content), 2 mL solvent, 7 h. Method A: Et_3_N, DMF, 100 °C, 30 bar. Method B: DBU, toluene, 120 °C, 5 bar. ^b^ Determined by GC. ^c^ After column chromatography. ^d^ Reaction time: 14 h. ^e^ Reaction time: 21 h.

**Table 4:** Aminocarbonylation of 6-iodoimidazo[1,2-*a*]pyridine (**1**) in the presence of aniline (**4f**) ^a^.

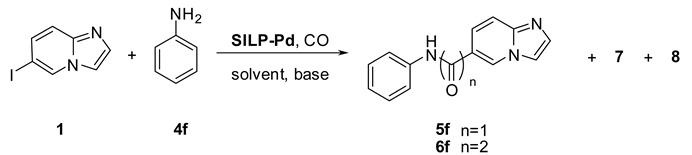									
Entry	Temp. [°C]	Pressure [bar]	R. Time [h]	Solvent	Base	Conv. [%] ^b^	Ratio of Products ^b^		
							5f [%]	6f [%]	7 + 8 [%]
1	100	30	3	DMF	Et_3_N	78	95	4	1
2	100	30	7	DMF	Et_3_N	99	92	4	4
3	100	50	7	DMF	Et_3_N	100	92	8	-
4	120	5	4	DMF	Et_3_N	83	97		3
5	120	5	7	DMF	Et_3_N	95	91	-	9
6	120	5	7	toluene	DBU	100	100	-	-

^a^ Reaction conditions: 0.2 mmol **1**, 0.5 mmol aniline (**4f**), 0.25 mmol base, catalyst **SILP-Pd** (with 2.8 μmol Pd-content), 2 mL solvent. ^b^ Determined by GC.

**Table 5:** Aminocarbonylation of 6-iodoimidazo[1,2-*a*]pyridine (**1**) in the presence of aromatic amines (**4f–i**) ^a^.

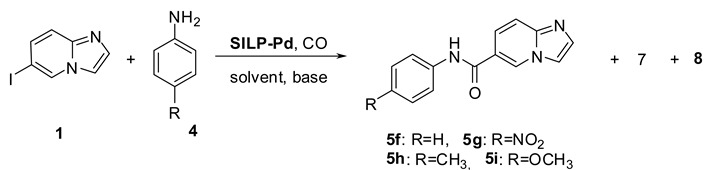							
Entry	Amine	R	Conv. [%] ^b^	Ratio of Products ^b^			Yield of 5 [%] ^c^
				5 [%]	6 [%]	7 + 8 [%]	
1	**4f**	H	95	91	-	9	84
2	**4g**	NO_2_	98	98	-	2	78
3	**4h**	CH_3_	100	100	-	-	82
4	**4i**	OCH_3_	99	97	-	-	64

^a^ Reaction conditions: 0.2 mmol **1**, 0.5 mmol amine (**4f–i**), 0.25 mmol Et_3_N, catalyst **SILP-Pd** (with 2.8 μmol Pd-content), 2 mL DMF, 120 °C, 5 bar, 7 h. ^b^ Determined by GC. ^c^ After column chromatography.

## Data Availability

Data are contained within the article or Supplementary Materials.

## Supplementary Materials

The following supporting information can be downloaded at: https://www.mdpi.com/article/10.3390/molecules29215048/s1, ^1^H- and ^13^C NMR spectra of iodoimidazo[1,2-*a*]pyridines **1**–**3** and products **5a**–**5i**, **6a**–**6e**, **9a**, **9f** and **10**.
