# Supplementary material for: Synthesis of 6- or 8-Carboxamido Derivatives of Imidazo[1,2-a]pyridines via a Heterogeneous Catalytic Aminocarbonylation Reaction
Source: Molecules. 2024 Oct 25;29(21):5048. doi: 10.3390/molecules29215048 (PMC11547779; doi:10.3390/molecules29215048)
Supplement: Supplementary file 1 [file molecules-29-05048-s001.zip › molecules-3278399-supplementary.pdf]

Supporting information

Synthesis of 6- or 8-Carboxamido Derivatives of Imidazo[1,2-*a*]pyridines via a Heterogeneous Catalytic Aminocarbonylation Reaction

Enikő Nagy [1], Attila Máriás [1], Margit Kovács [2] and Rita Skoda-Földes [1,*]

[1] Research Group of Organic Synthesis and Catalysis, University of Pannonia, Egyetem u. 10, 8200 Veszprém, Hungary; nagyencsi6@gmail.com (E.N.); marias.attila2000@gmail.com (A.M.)
[2] NMR Laboratory, University of Pannonia, Egyetem u. 10, 8200 Veszprém, Hungary; kovacs.margit@mk.uni-pannon.hu
* Correspondence: skodane.foldes.rita@mk.uni-pannon.hu

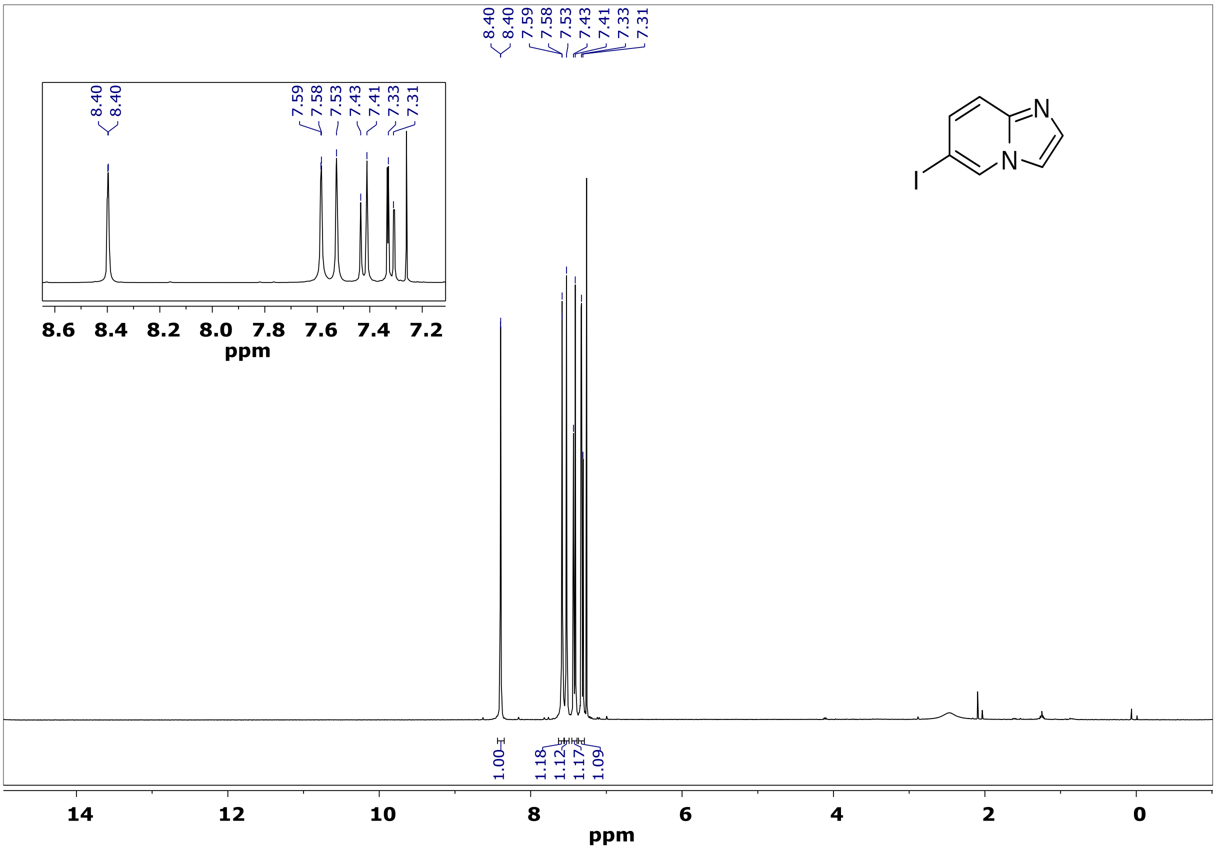

^{1}H NMR spectrum of 6-iodo-imidazo[1,2-*a*]pyridine (**1**) ($CDCl_3$)

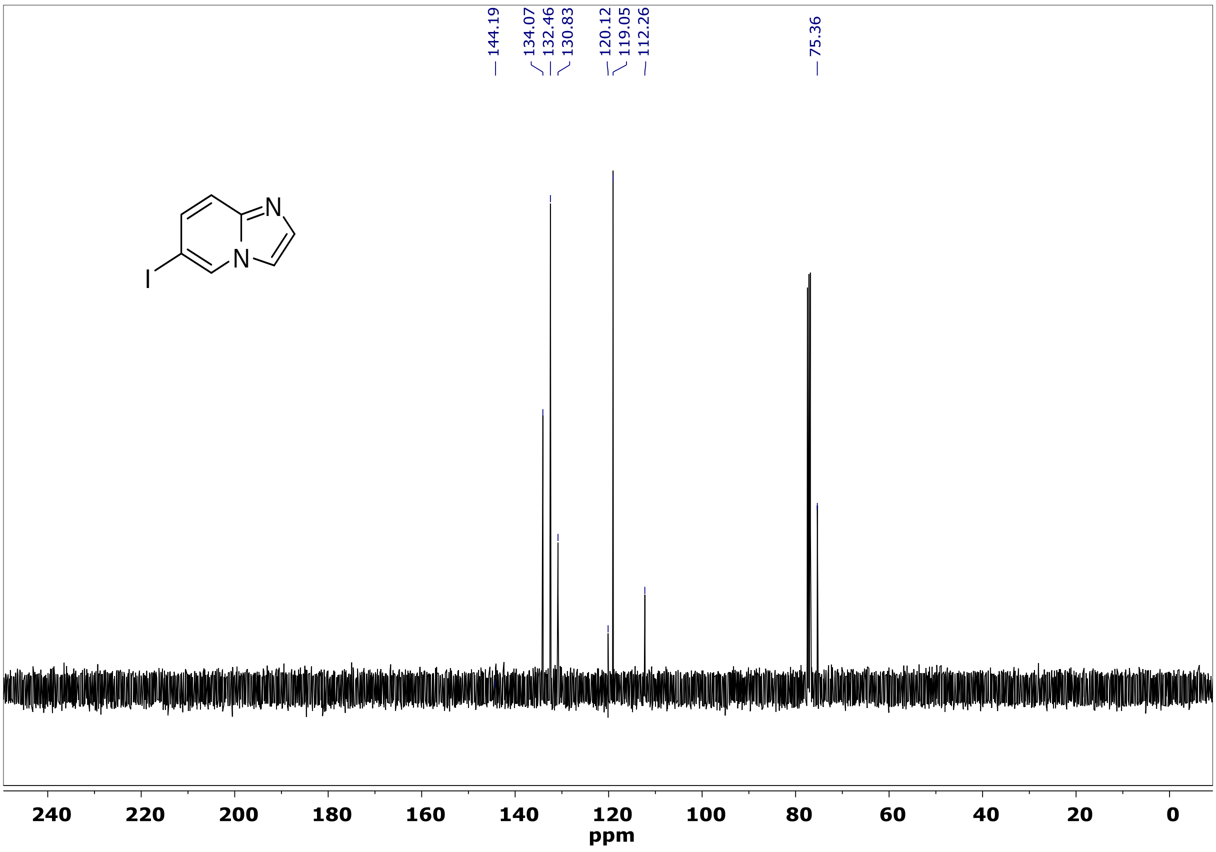

^{13}C{^{1}H} NMR spectrum of 6-iodo-imidazo[1,2-*a*]pyridine (**1**) ($CDCl_3$)

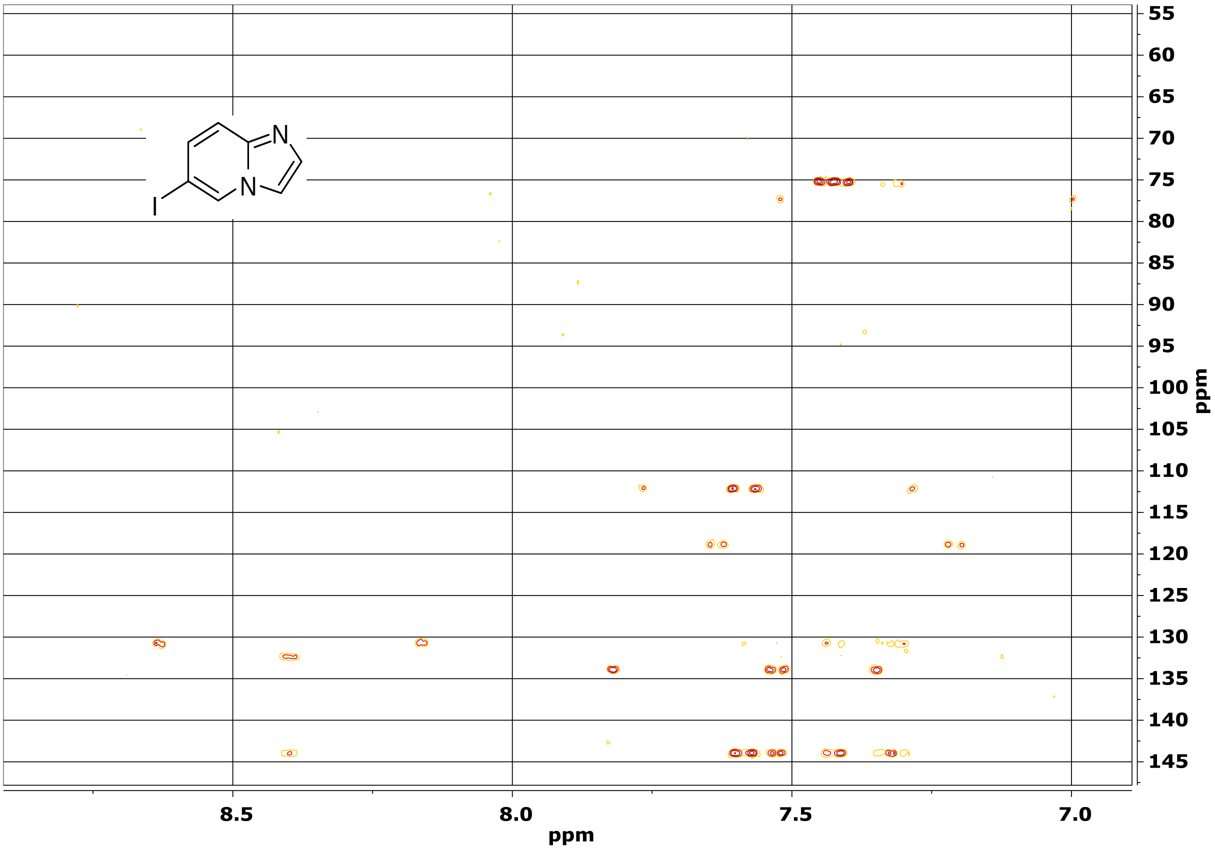

HMBC spectrum of 6-iodo-imidazo[1,2-*a*]pyridine (**1**) ($CDCl_3$)

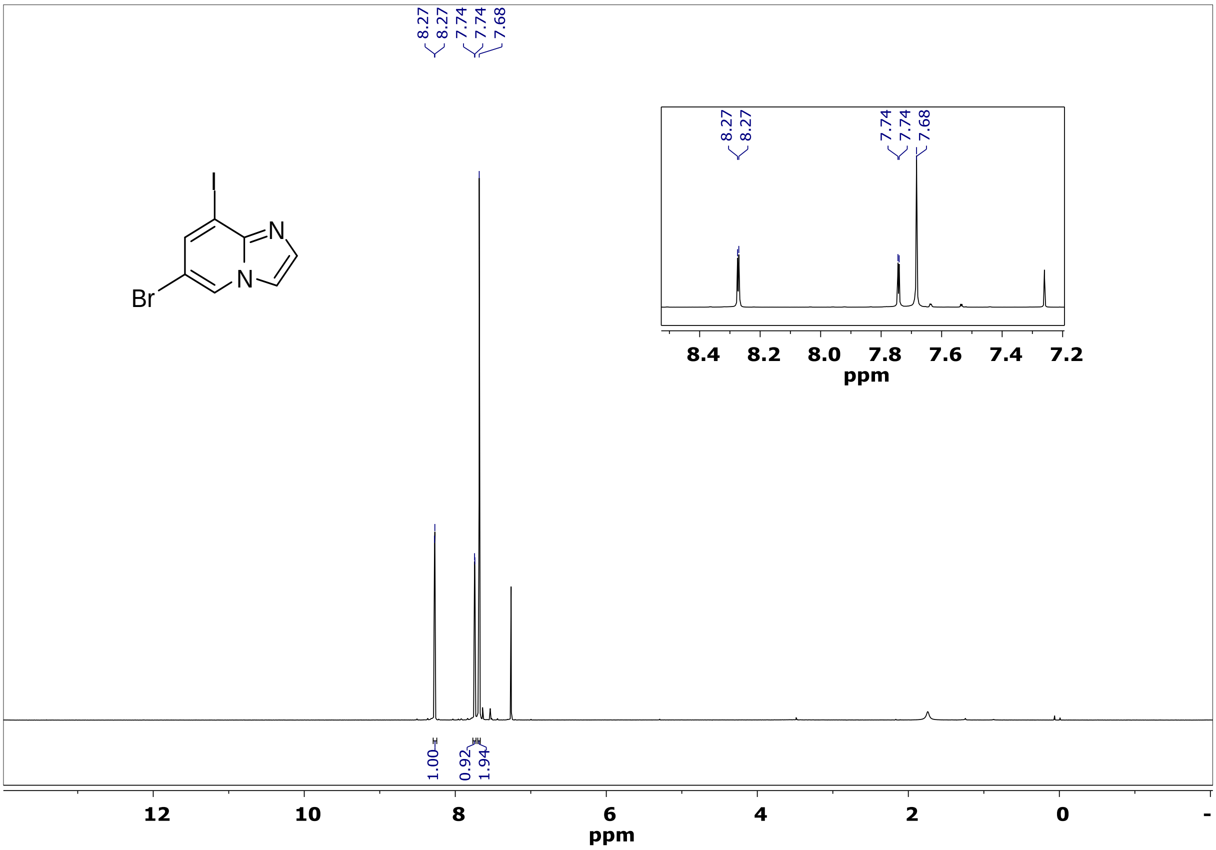

^{1}H NMR spectrum of 6-bromo-8-iodo-imidazo[1,2-*a*]pyridine (**2**) ($CDCl_3$)

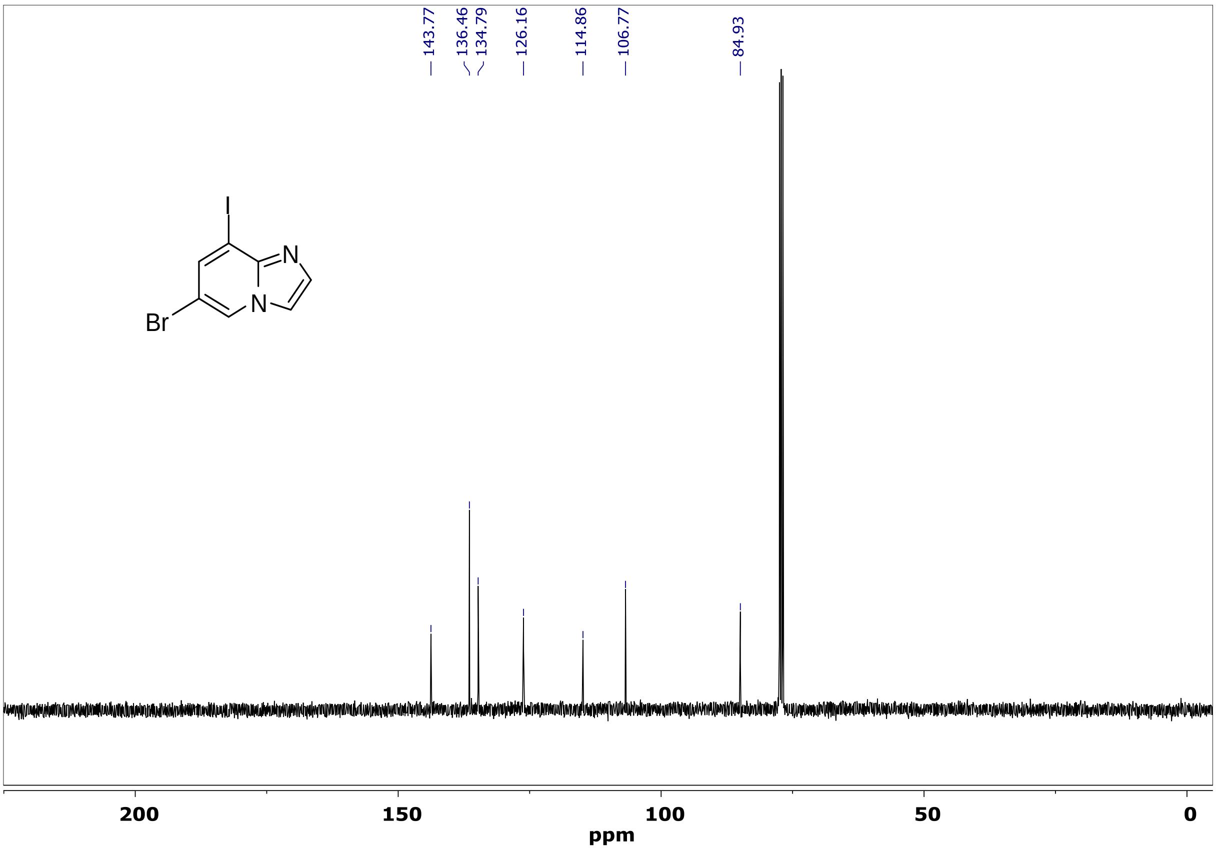

^{13}C{^{1}H} NMR spectrum of 6-bromo-8-iodo-imidazo[1,2-*a*]pyridine (**2**) ($CDCl_3$)

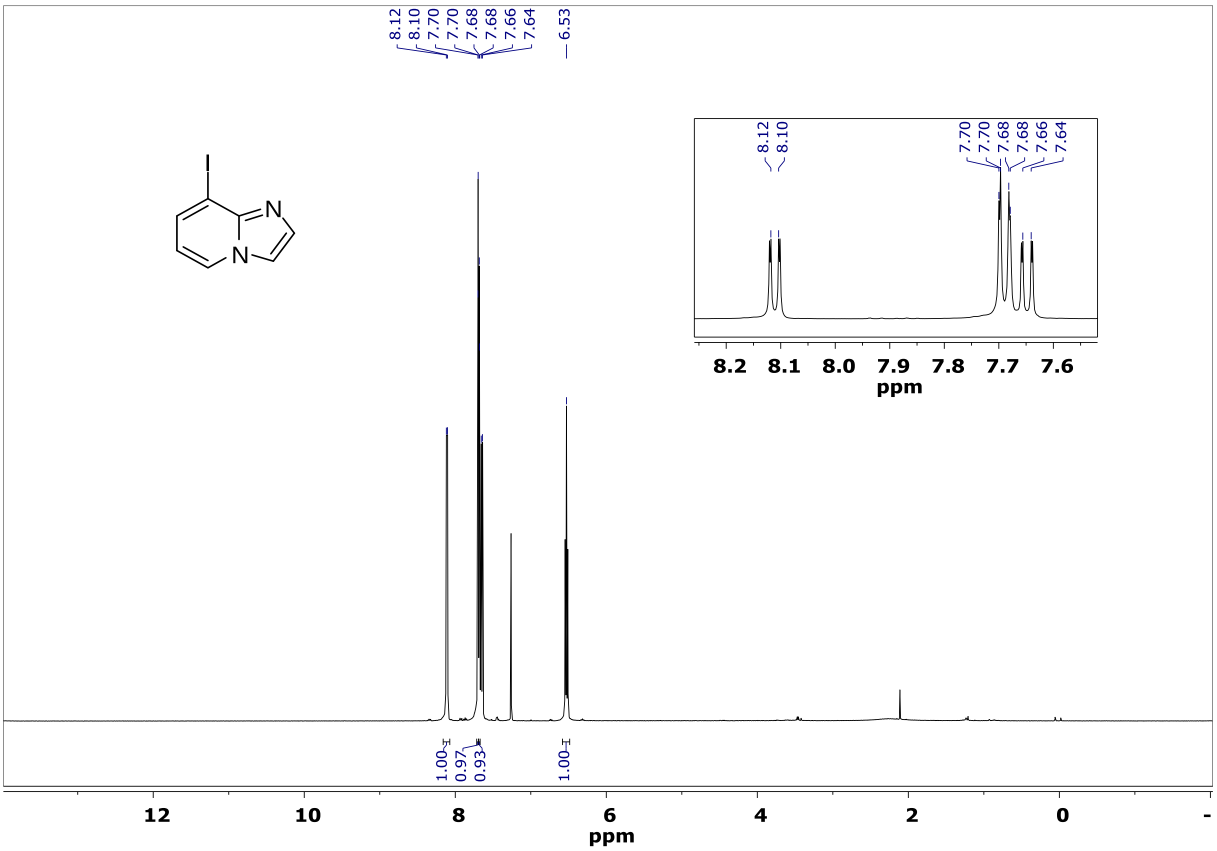

^{1}H NMR spectrum of 8-iodo-imidazo[1,2-*a*]pyridine (**3**) ($CDCl_3$)

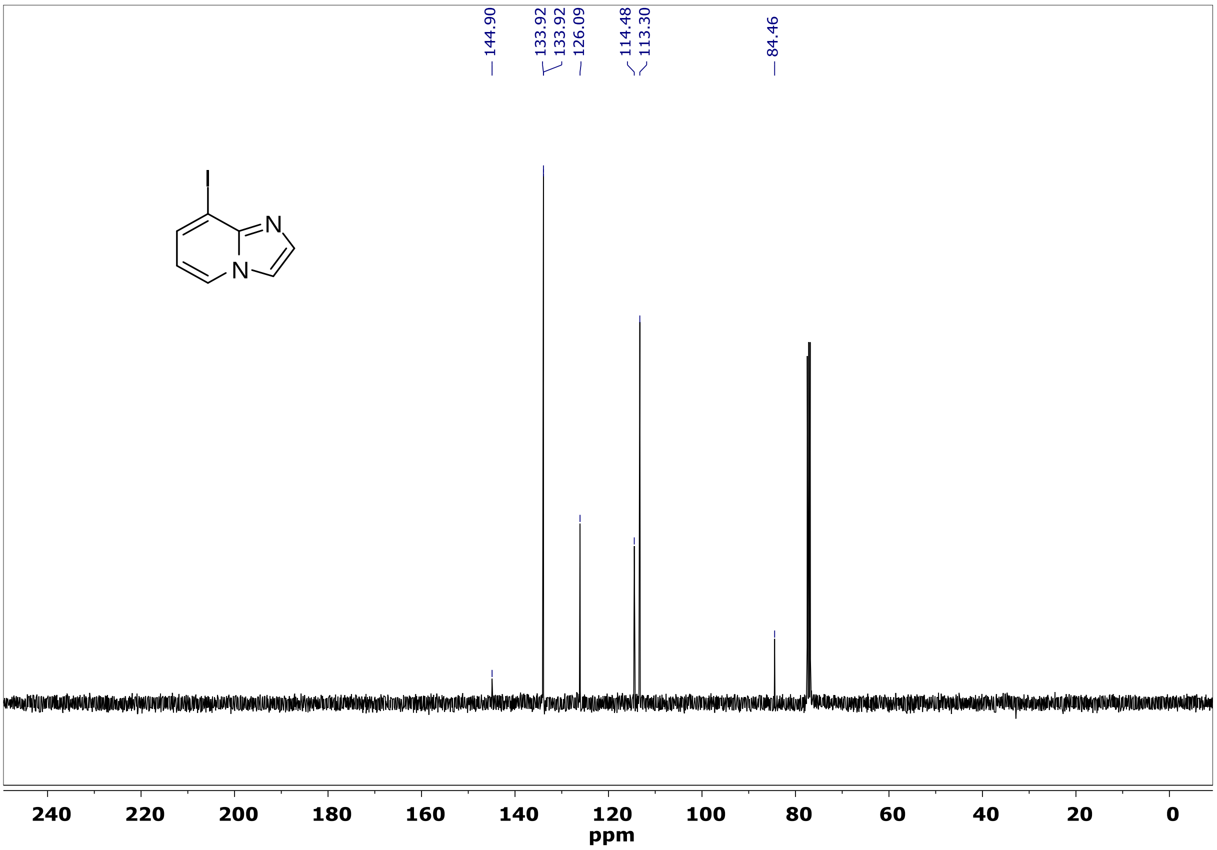

$^{13}C\{^{1}H\}$ NMR spectrum of 8-iodo-imidazo[1,2-*a*]pyridine (**3**) ($CDCl_3$)

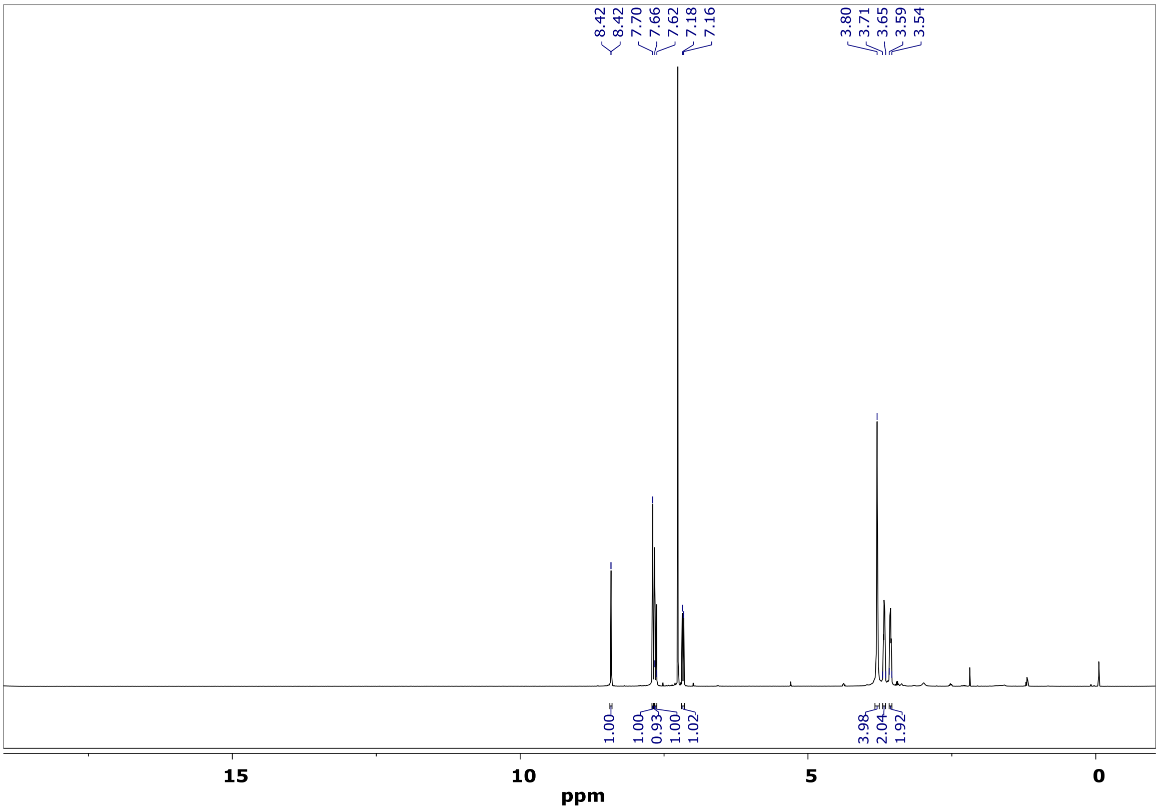

^{1}H NMR spectrum of imidazo[1,2-*a*]pyridin-6-yl(morpholino)methanone (**5a**) ($CDCl_3$, 227K)

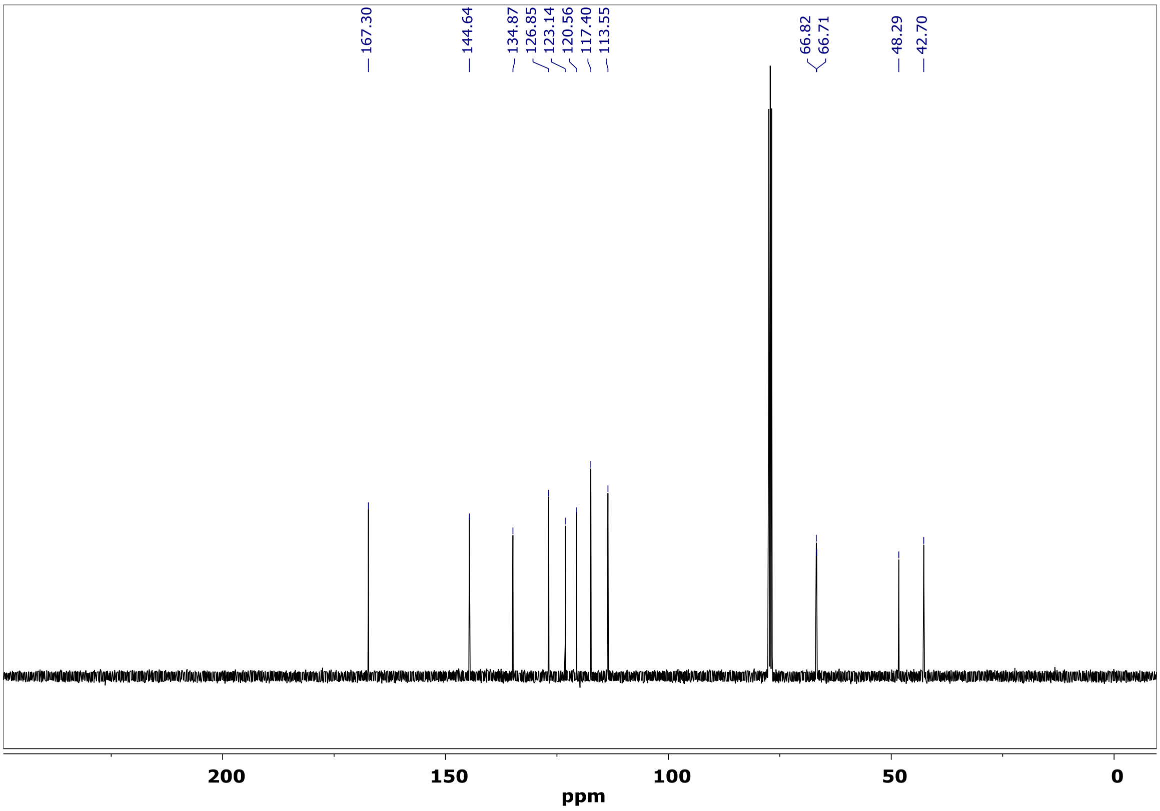

^{13}C{^{1}H} NMR spectrum of imidazo[1,2-*a*]pyridin-6-yl(morpholino)methanone (**5a**) ($CDCl_3$, 227K)

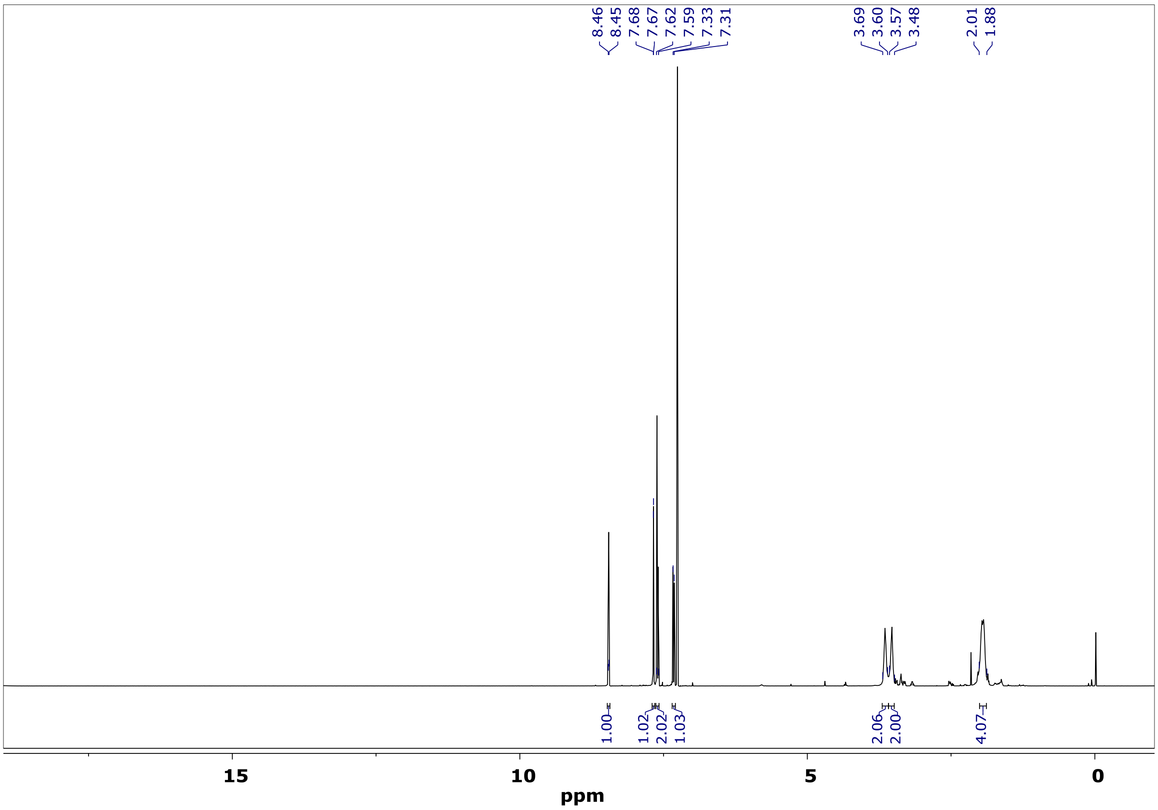

^{1}H NMR spectrum of imidazo[1,2-*a*]pyridin-6-yl(pyrrolidin-1-yl)methanone (**5b**) ($CDCl_3$)

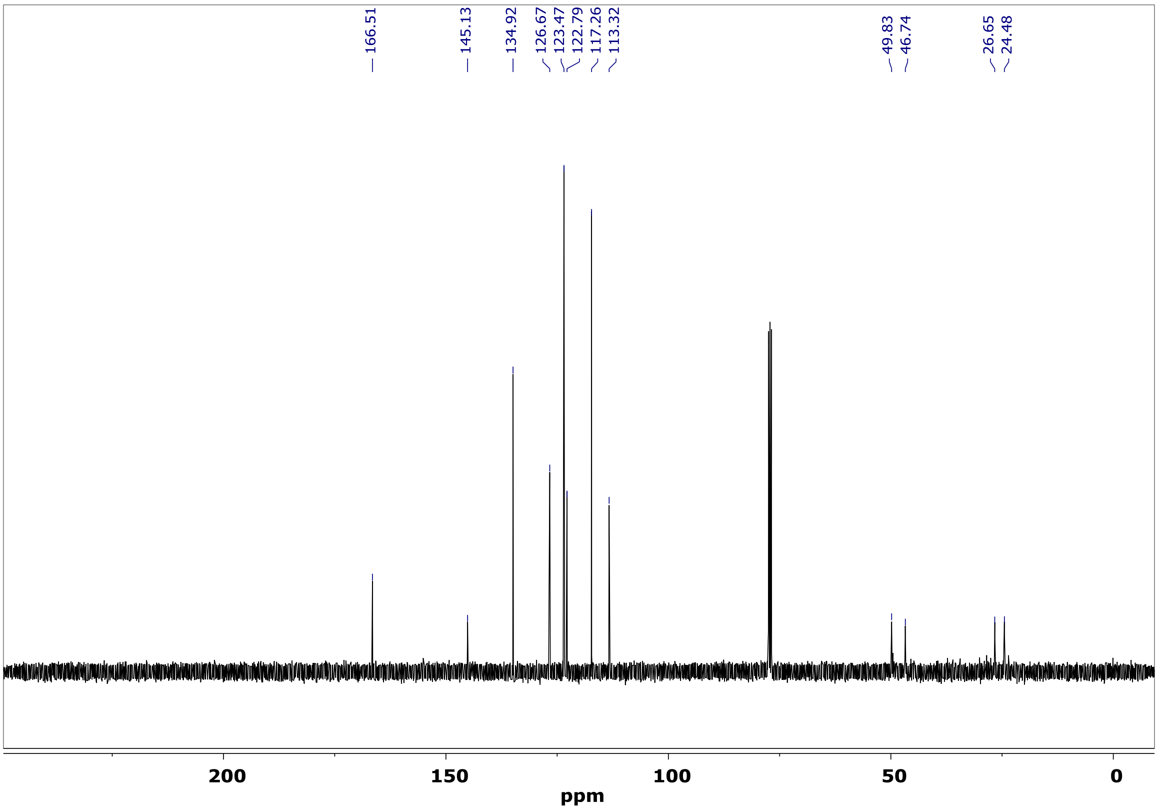

^{13}C{^{1}H} NMR spectrum of imidazo[1,2-*a*]pyridin-6-yl(pyrrolidin-1-yl)methanone (**5b**) ($CDCl_3$, 227K)

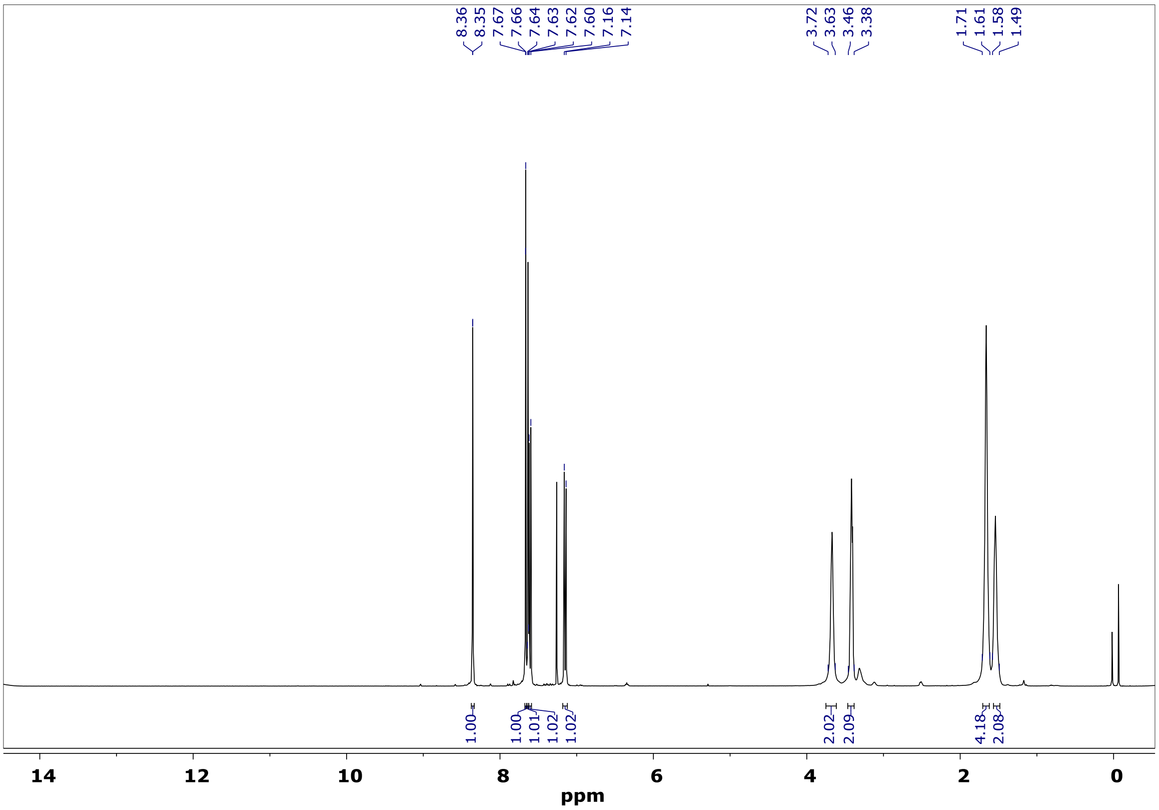

^{1}H NMR spectrum of imidazo[1,2-*a*]pyridin-6-yl(piperidin-1-yl)methanone (**5c**) ($CDCl_3$, 233K)

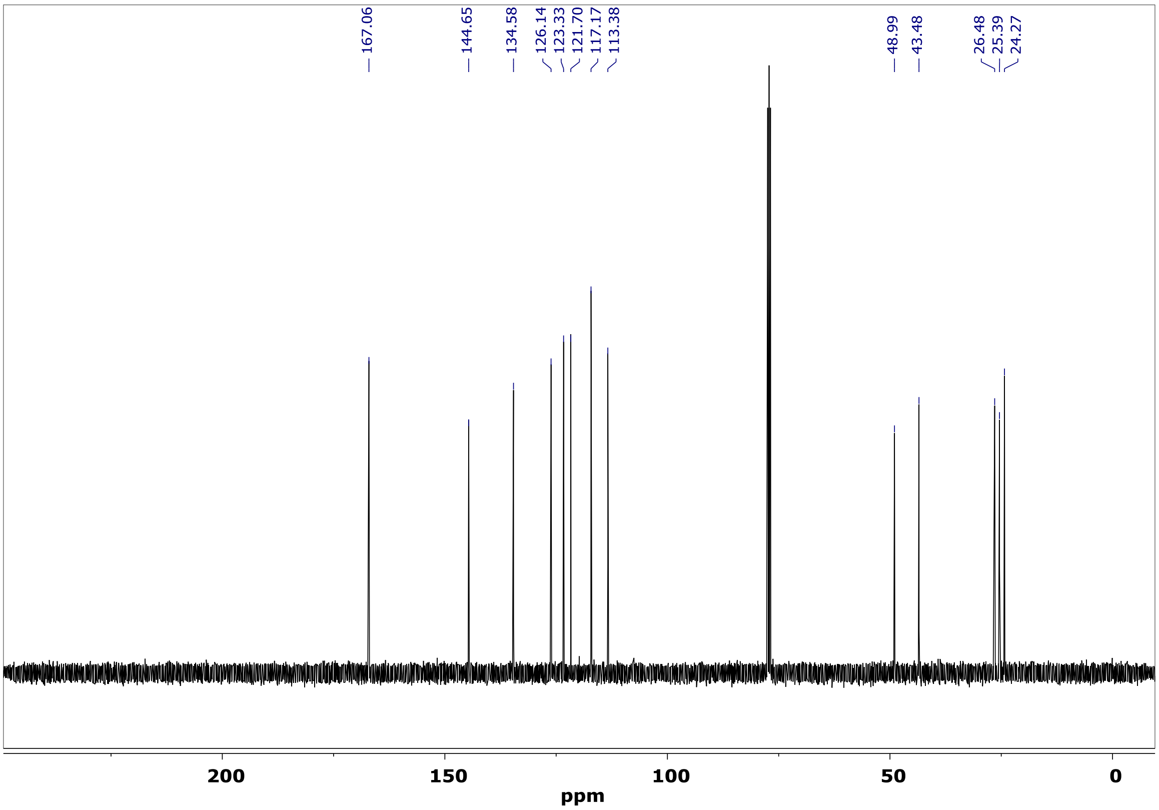

^{13}C{^{1}H} NMR spectrum of imidazo[1,2-*a*]pyridin-6-yl(piperidin-1-yl)methanone (**5c**) ($CDCl_3$, 233K)

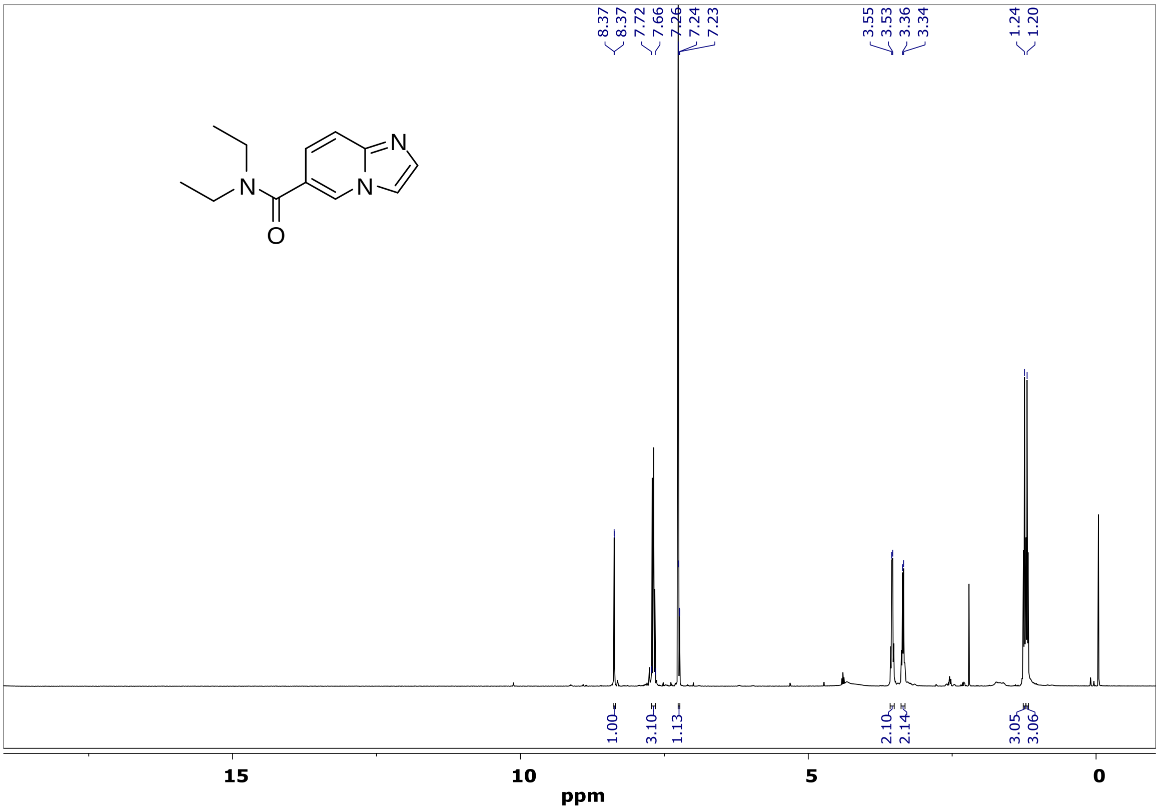

^{1}H NMR spectrum of *N,N*-diethylimidazo[1,2-*a*]pyridine-6-carboxamide (**5d**) ($CDCl_3$)

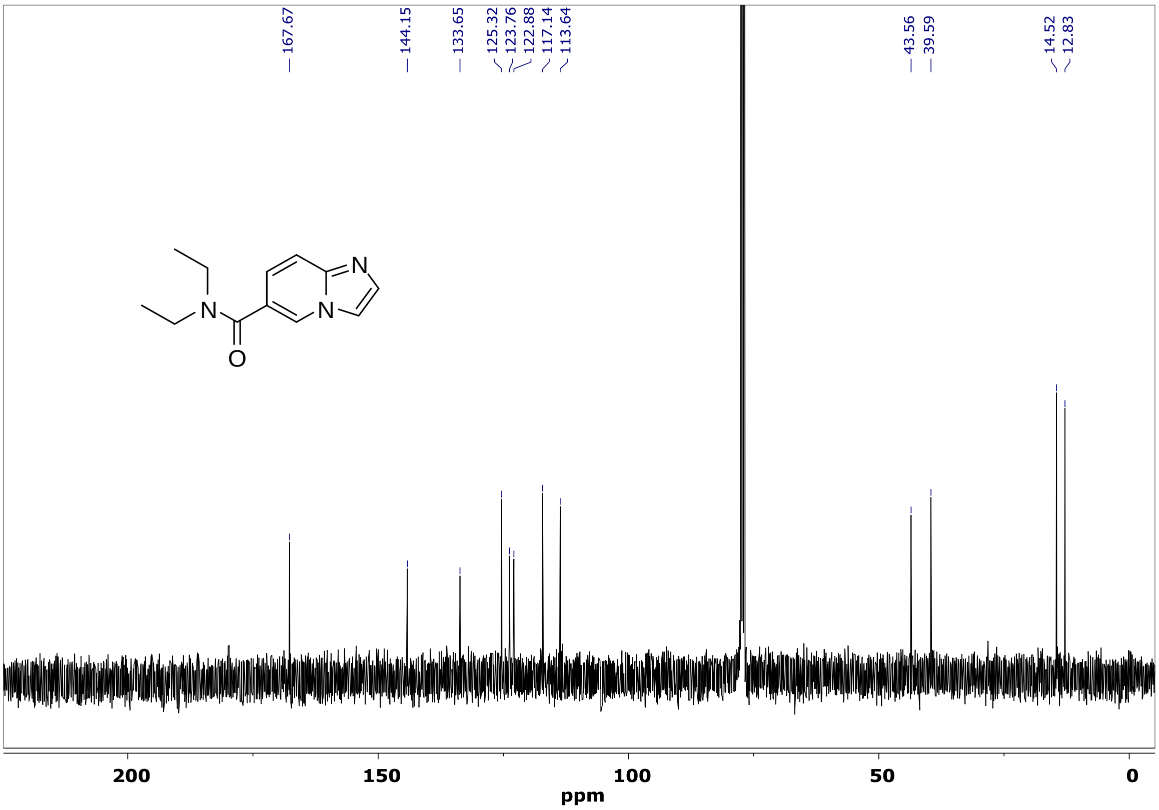

^{13}C{^{1}H} NMR spectrum of *N,N*-diethylimidazo[1,2-*a*]pyridine-6-carboxamide (**5d**) ($CDCl_3$, 227 K)

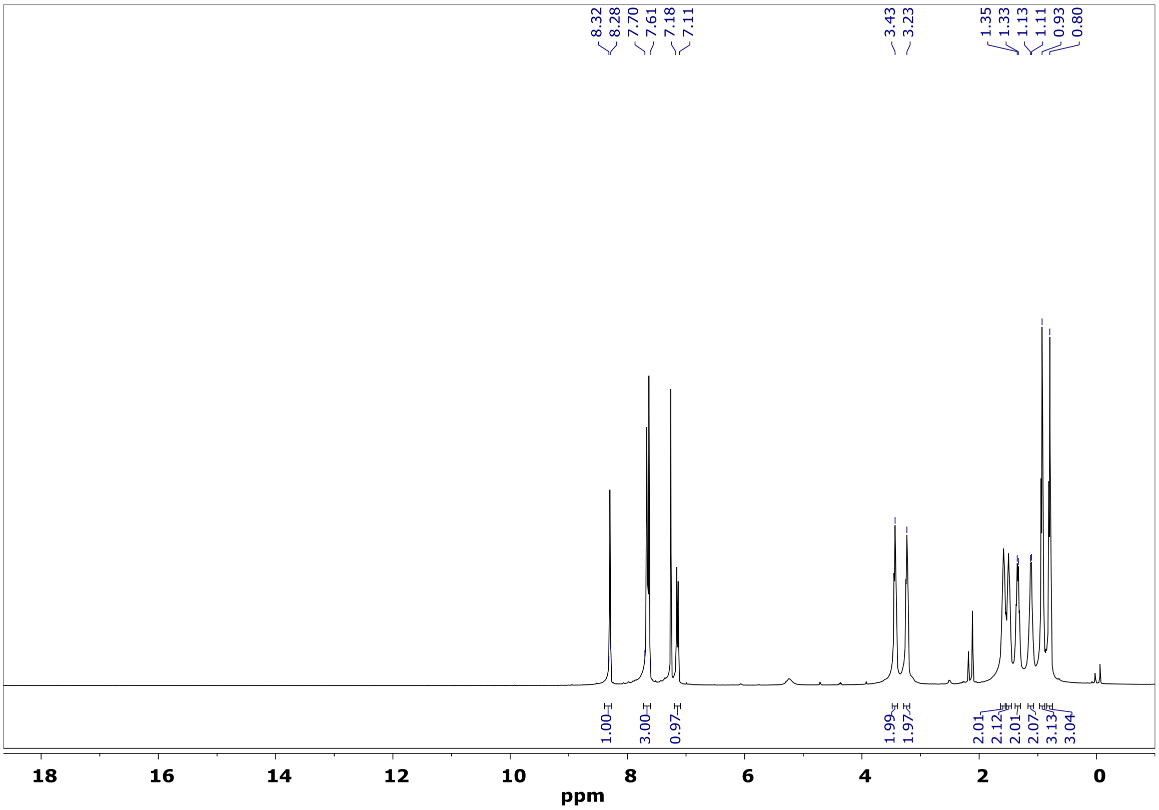

^{1}H NMR spectrum of *N,N*-dibutylimidazo[1,2-*a*]pyridine-6-carboxamide (**5e**) ($CDCl_3$, 227 K)

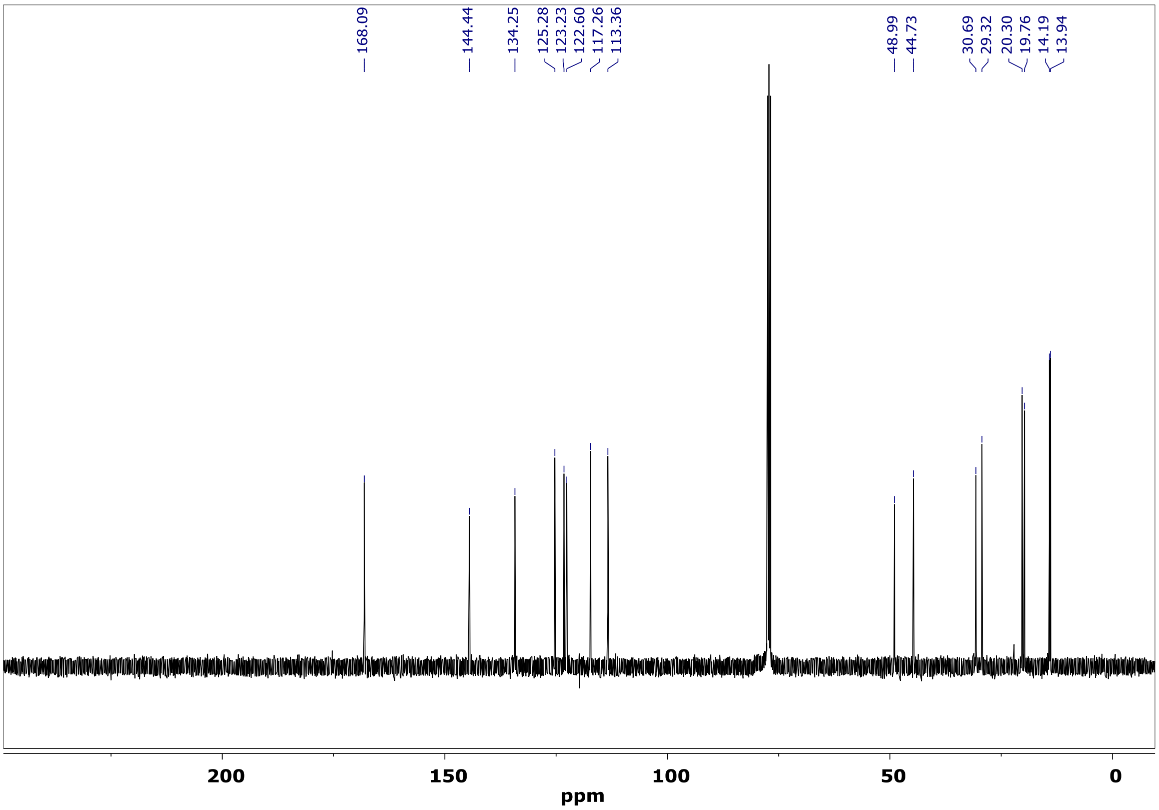

^{13}C{^{1}H} NMR spectrum of *N,N*-dibutylimidazo[1,2-*a*]pyridine-6-carboxamide (**5e**) ($CDCl_3$, 227 K)

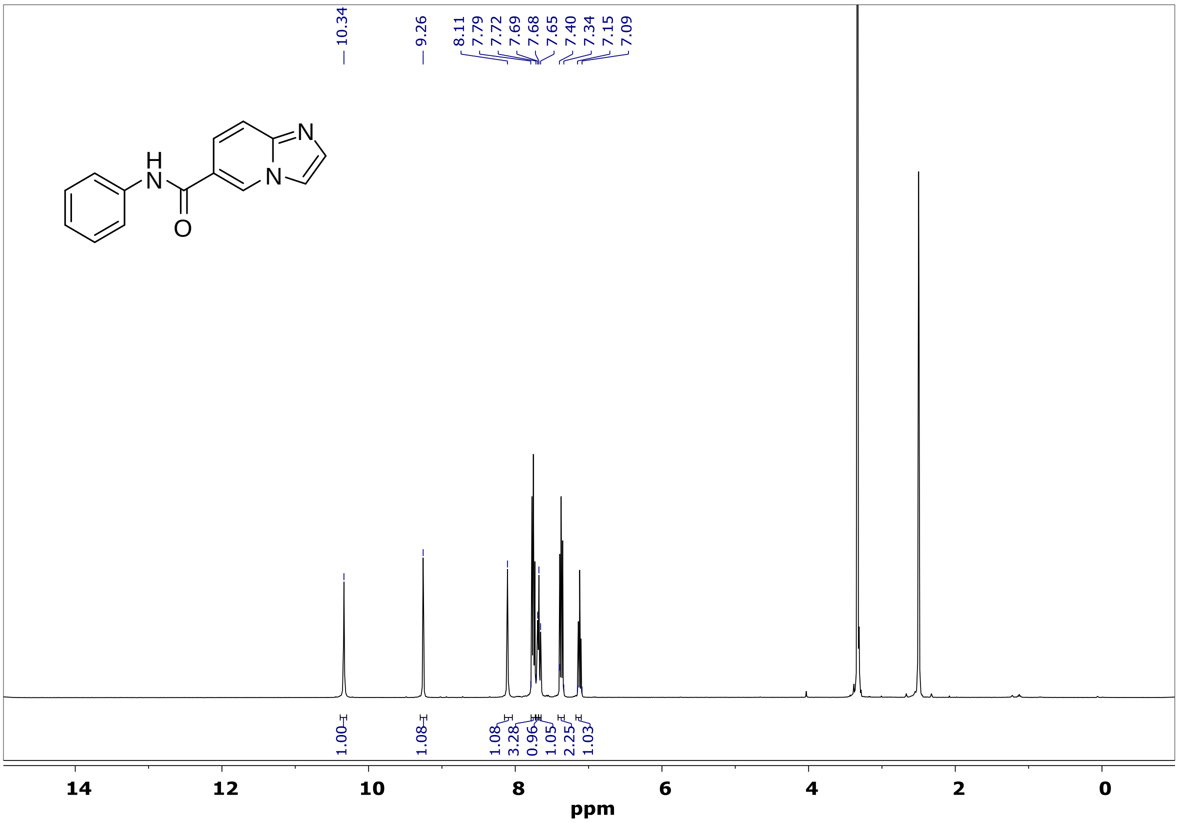

^{1}H NMR spectrum of *N*-phenylimidazo[1,2-*a*]pyridine-6-carboxamide (**5f**) (DMSO-d$_6$)

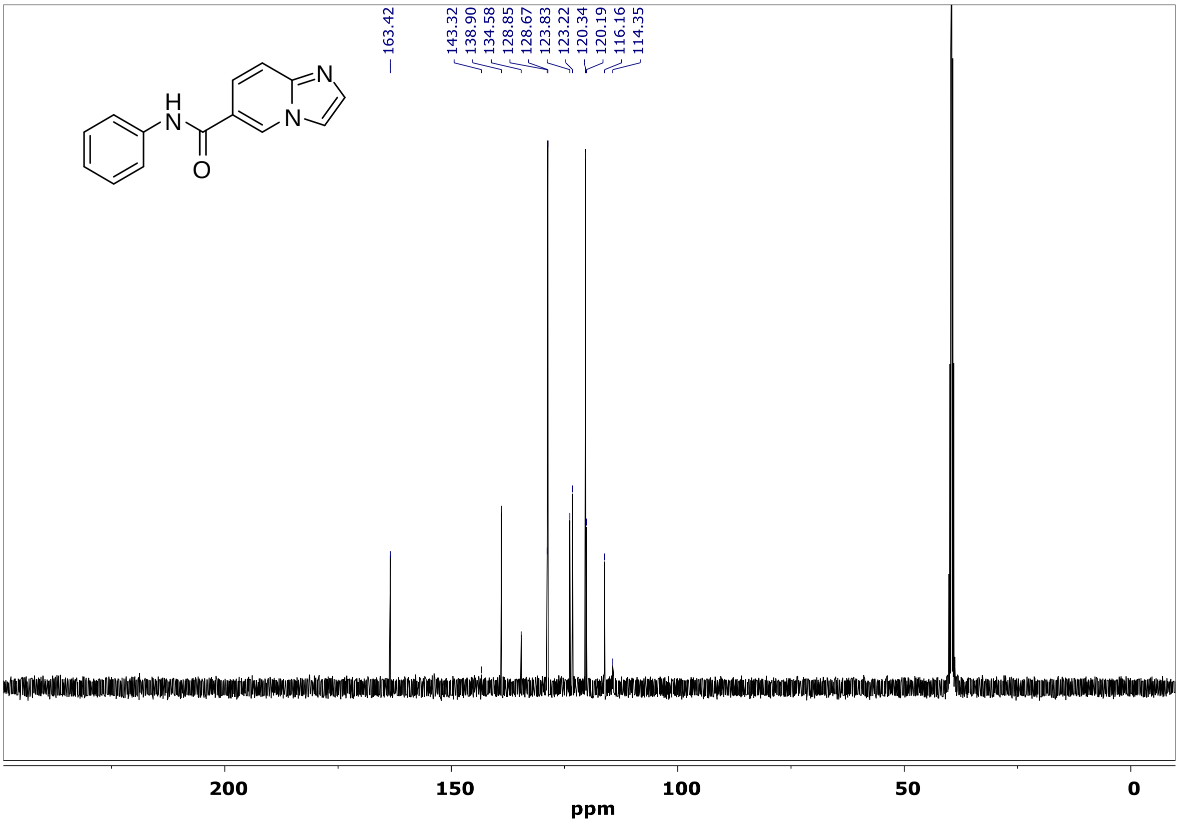

^{13}C{^{1}H} NMR spectrum of *N*-phenylimidazo[1,2-*a*]pyridine-6-carboxamide (**5f**) (DMSO-d$_6$)

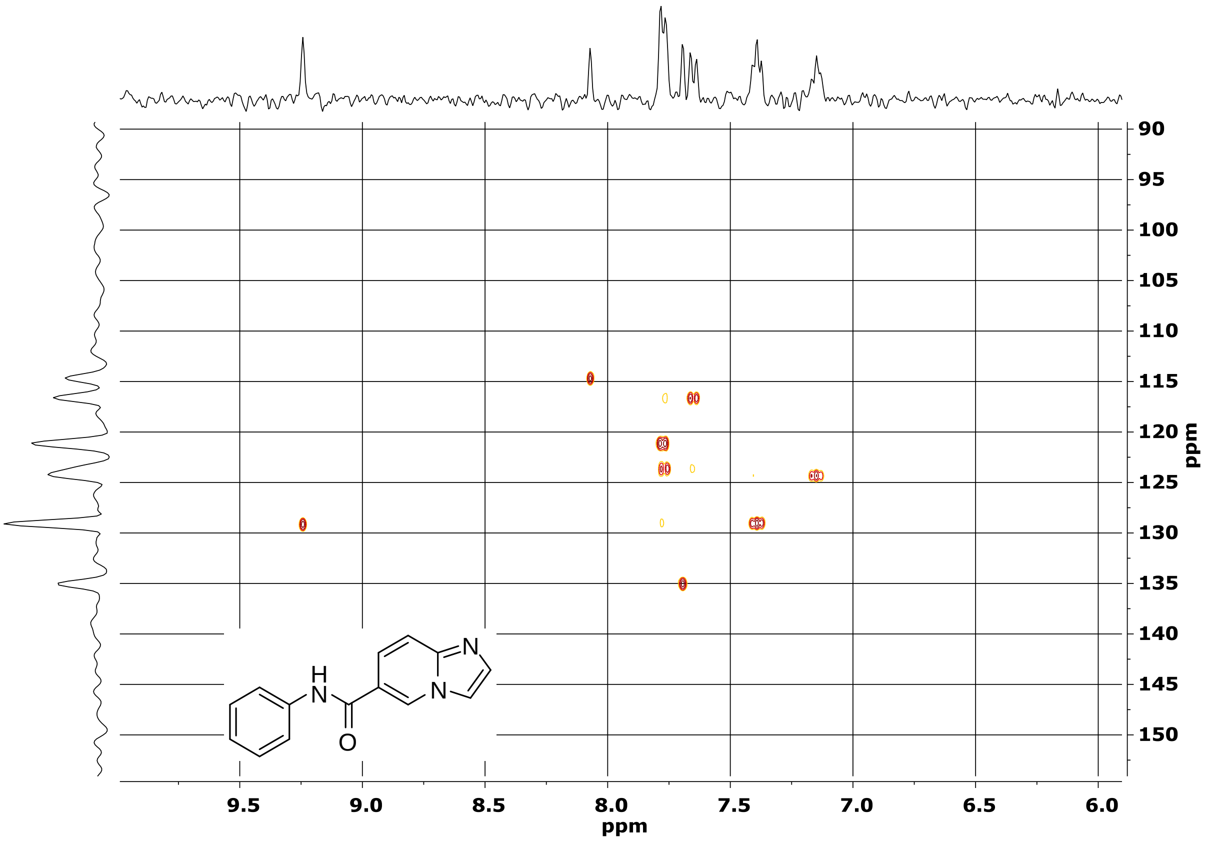

HSQC spectrum of *N*-phenylimidazo[1,2-*a*]pyridine-6-carboxamide (**5f**) (DMSO-d$_6$)

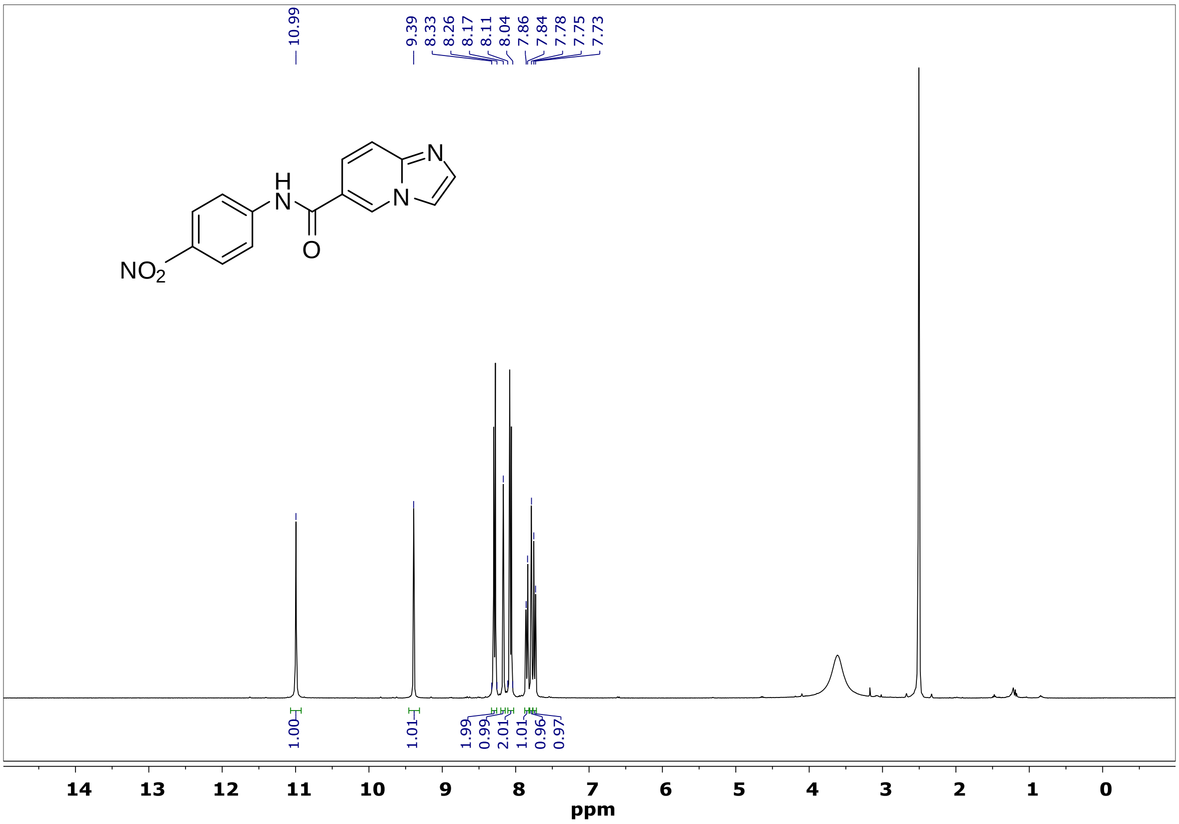

^{1}H NMR spectrum of *N*-(4-nitrophenyl)imidazo[1,2-*a*]pyridine-6-carboxamide (**5g**) (DMSO-d6)

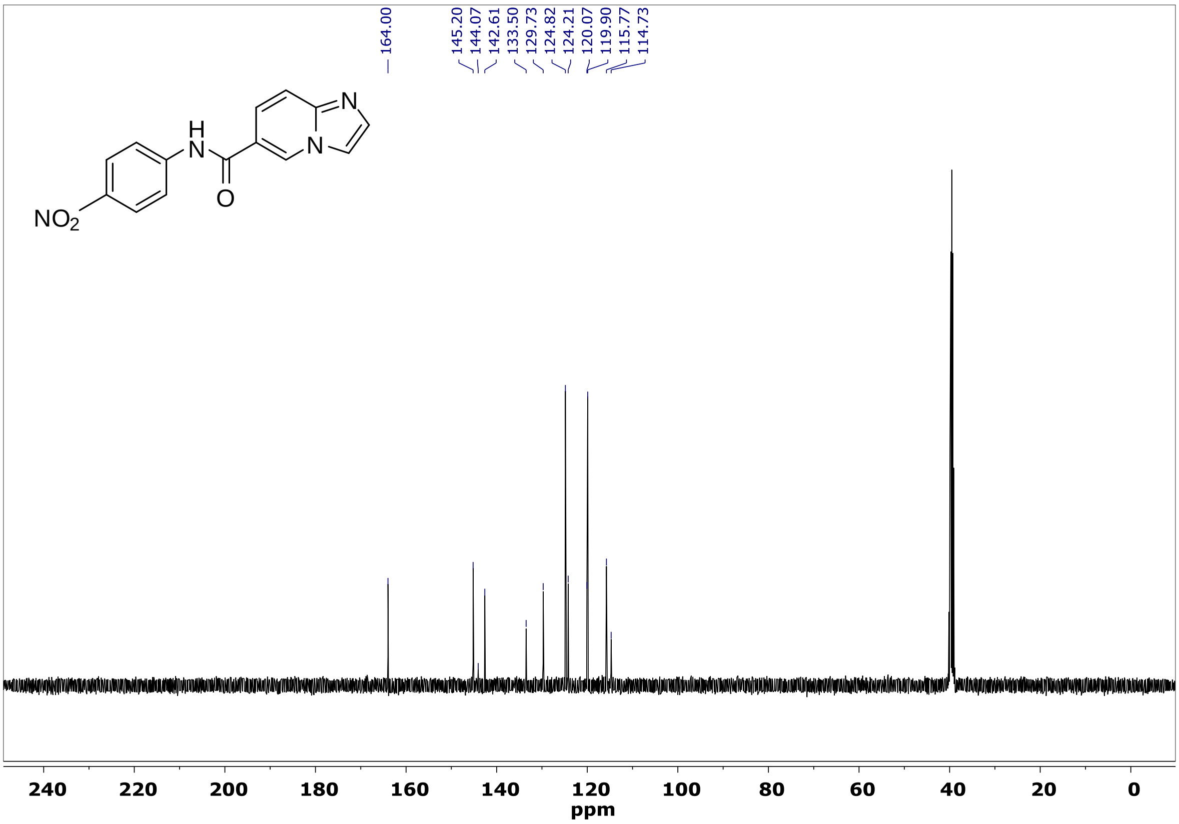

^{13}C{^{1}H} NMR spectrum of *N*-(4-nitrophenyl)imidazo[1,2-*a*]pyridine-6-carboxamide (**5g**) (DMSO-d6)

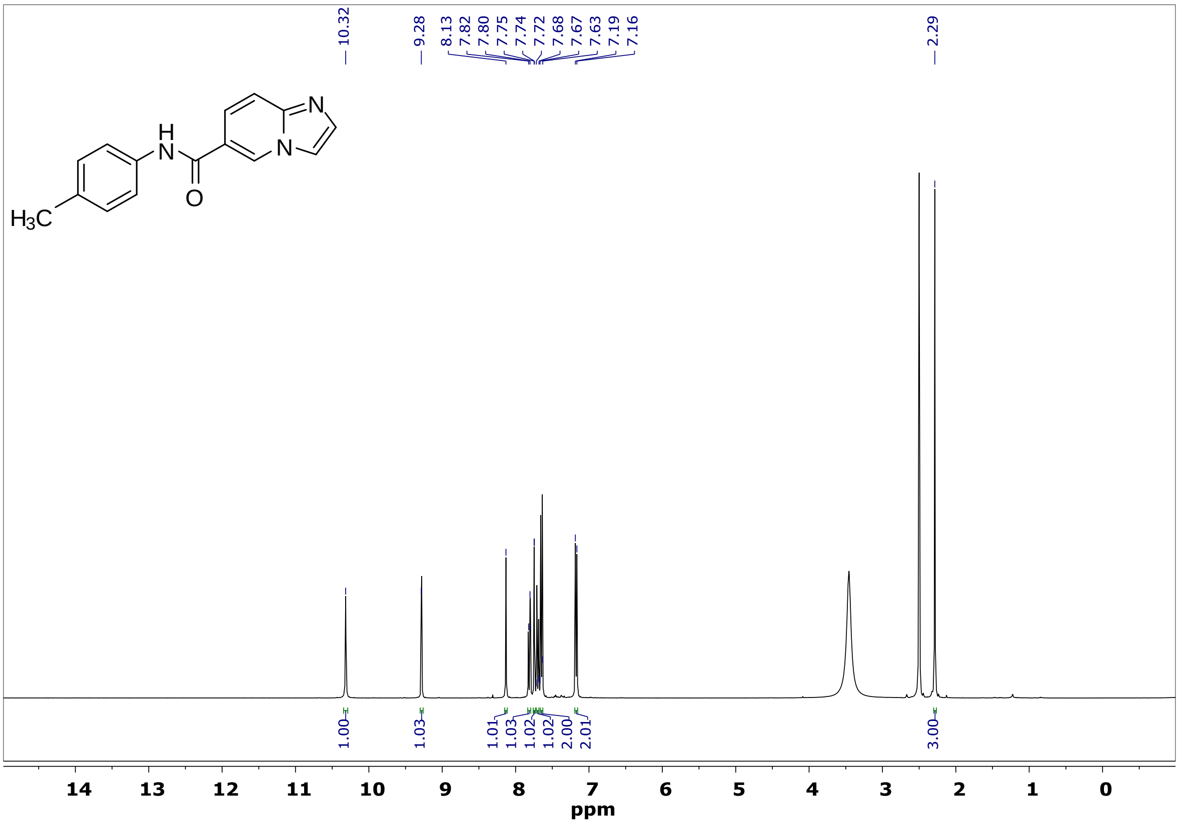

^{1}H NMR spectrum of *N*-(4-methylphenyl)imidazo[1,2-*a*]pyridine-6-carboxamide (**5h**) (DMSO-d$_6$)

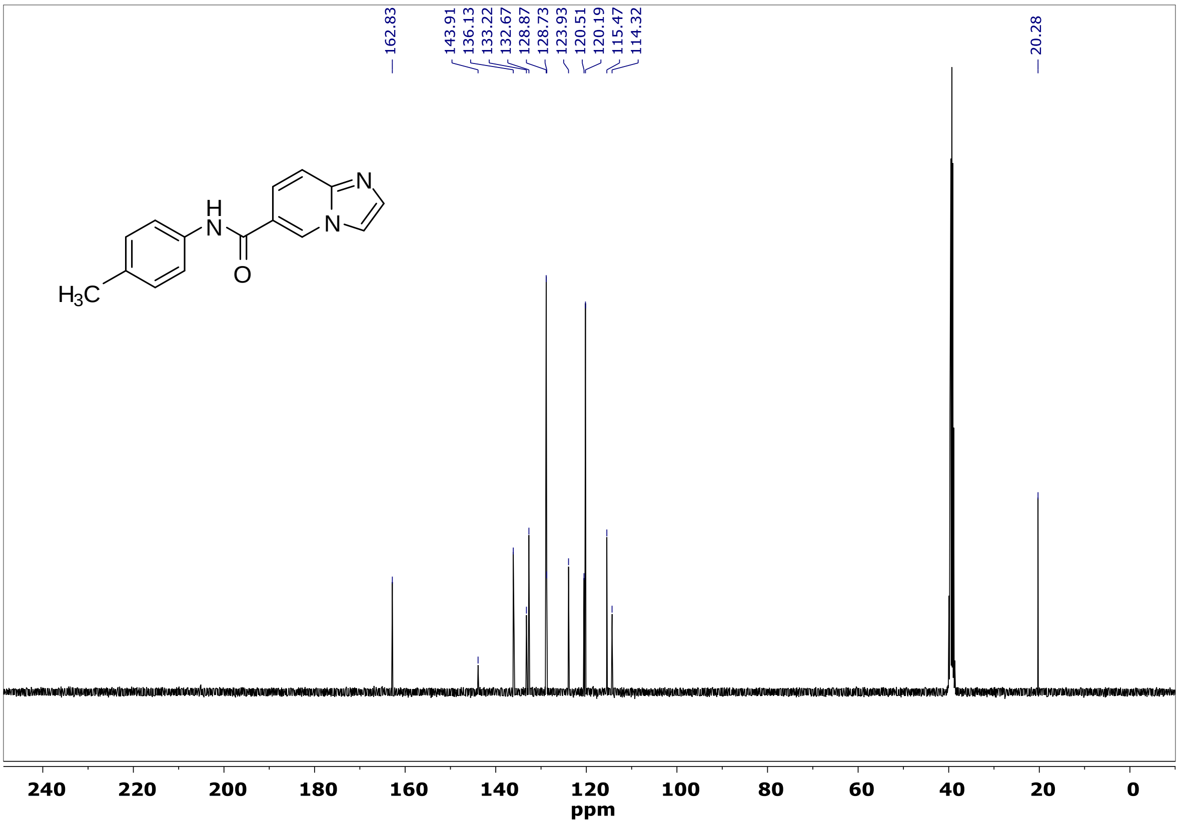

^{13}C{^{1}H} NMR spectrum of *N*-(4-methylphenyl)imidazo[1,2-*a*]pyridine-6-carboxamide (**5h**) (DMSO-d$_6$)

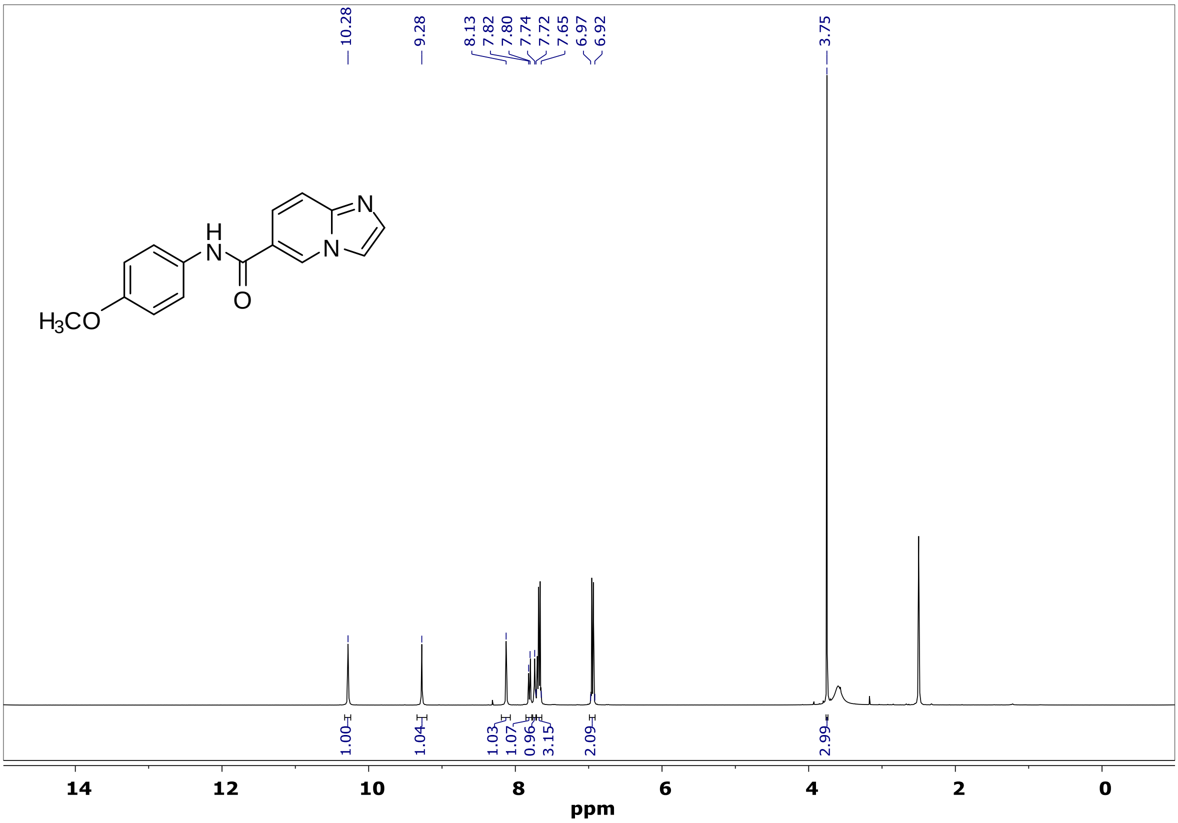

^{1}H NMR spectrum of *N*-(4-methoxyphenyl)imidazo[1,2-*a*]pyridine-6-carboxamide (**5i**) (DMSO-d6)

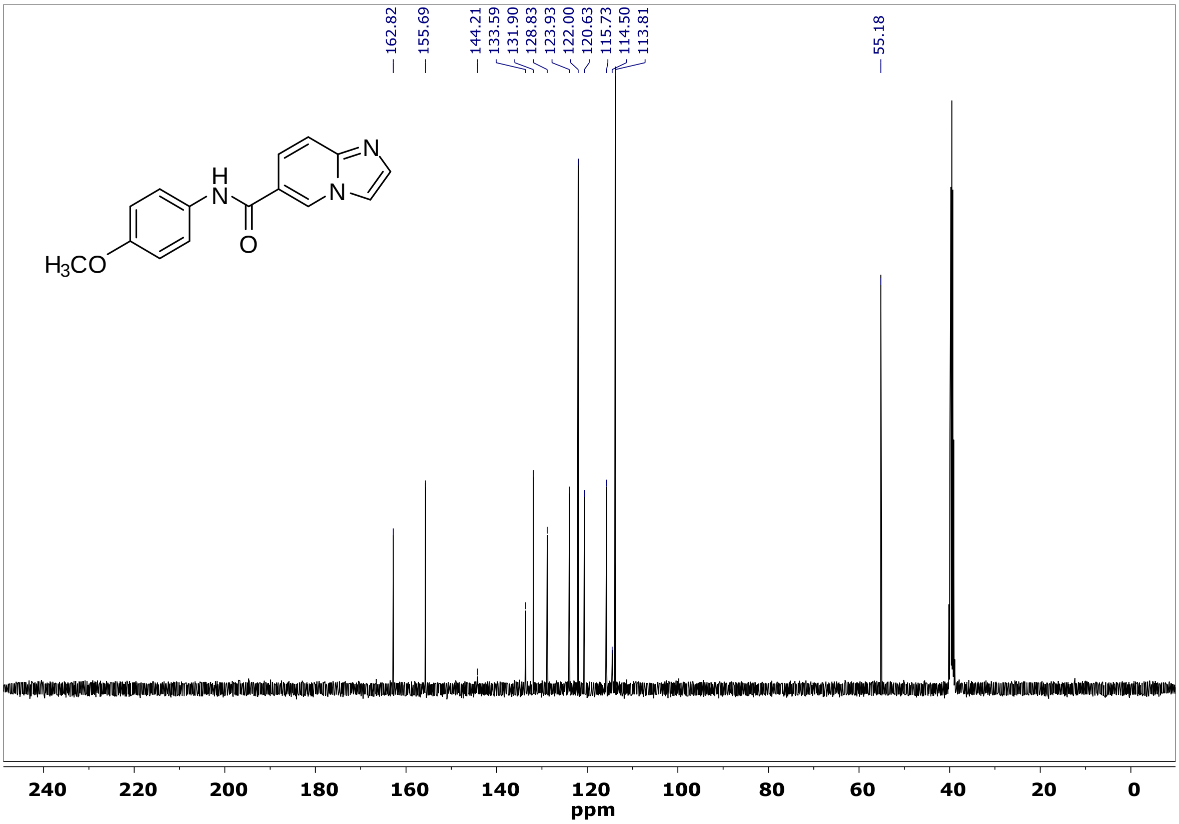

^{13}C{^{1}H} NMR spectrum of *N*-(4-methoxyphenyl)imidazo[1,2-*a*]pyridine-6-carboxamide (**5i**) (DMSO-d6)

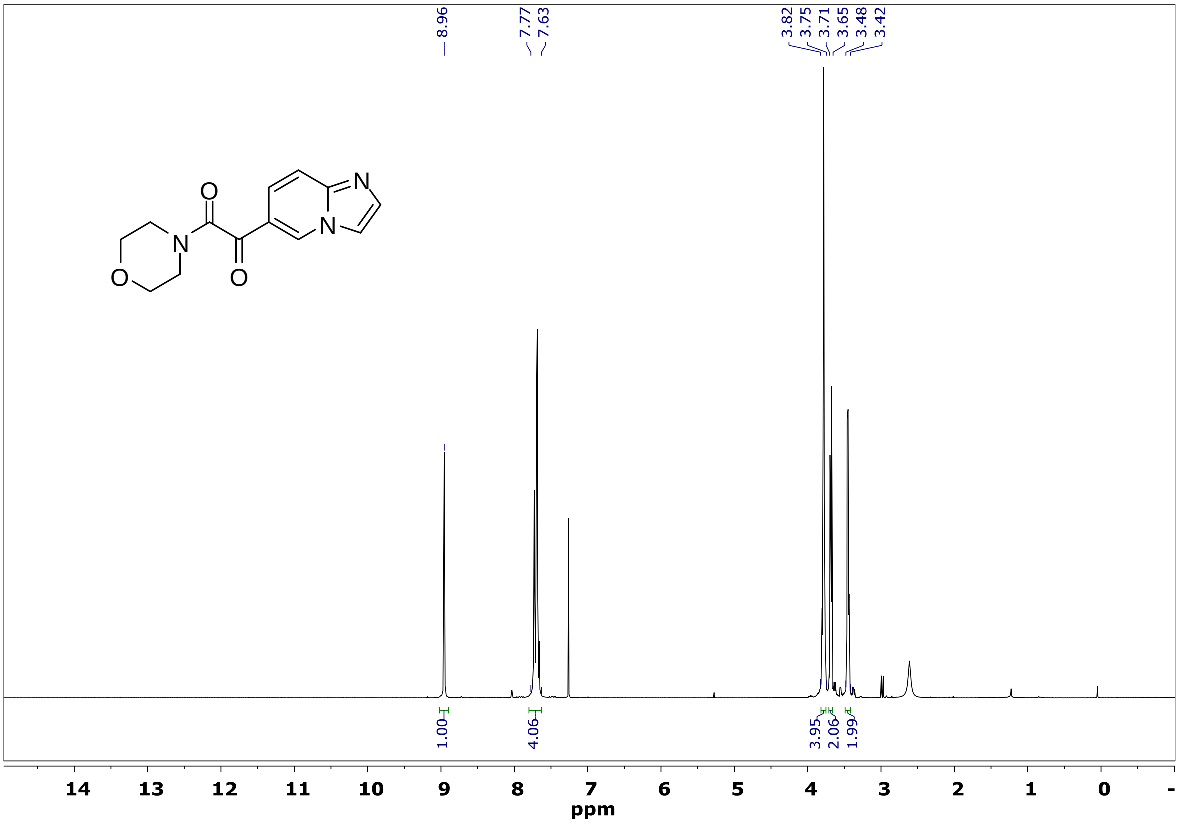

^{1}H NMR spectrum of 1-(imidazo[1,2-*a*]pyridin-6-yl)-2-morpholinoethane-1,2-dione (**6a**) ($CDCl_3$)

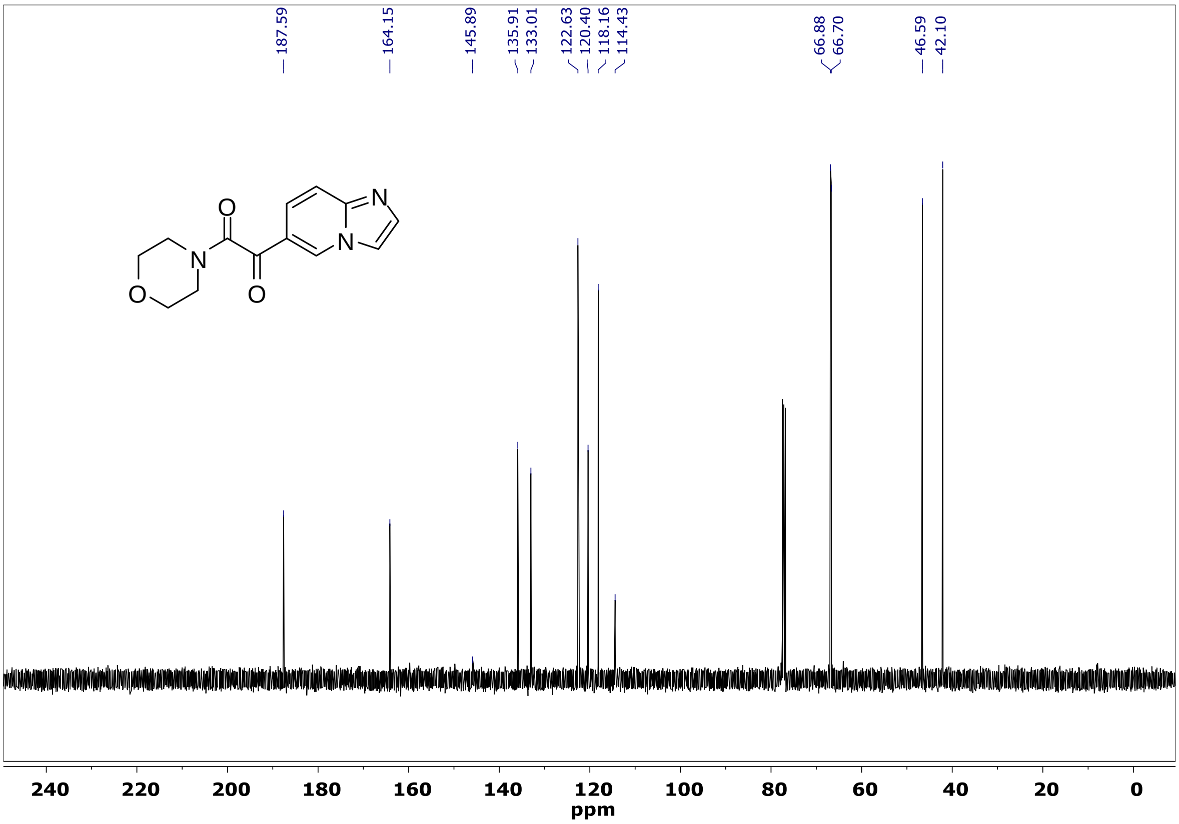

^{13}C{^{1}H} NMR spectrum of 1-(imidazo[1,2-*a*]pyridin-6-yl)-2-morpholinoethane-1,2-dione (**6a**) ($CDCl_3$)

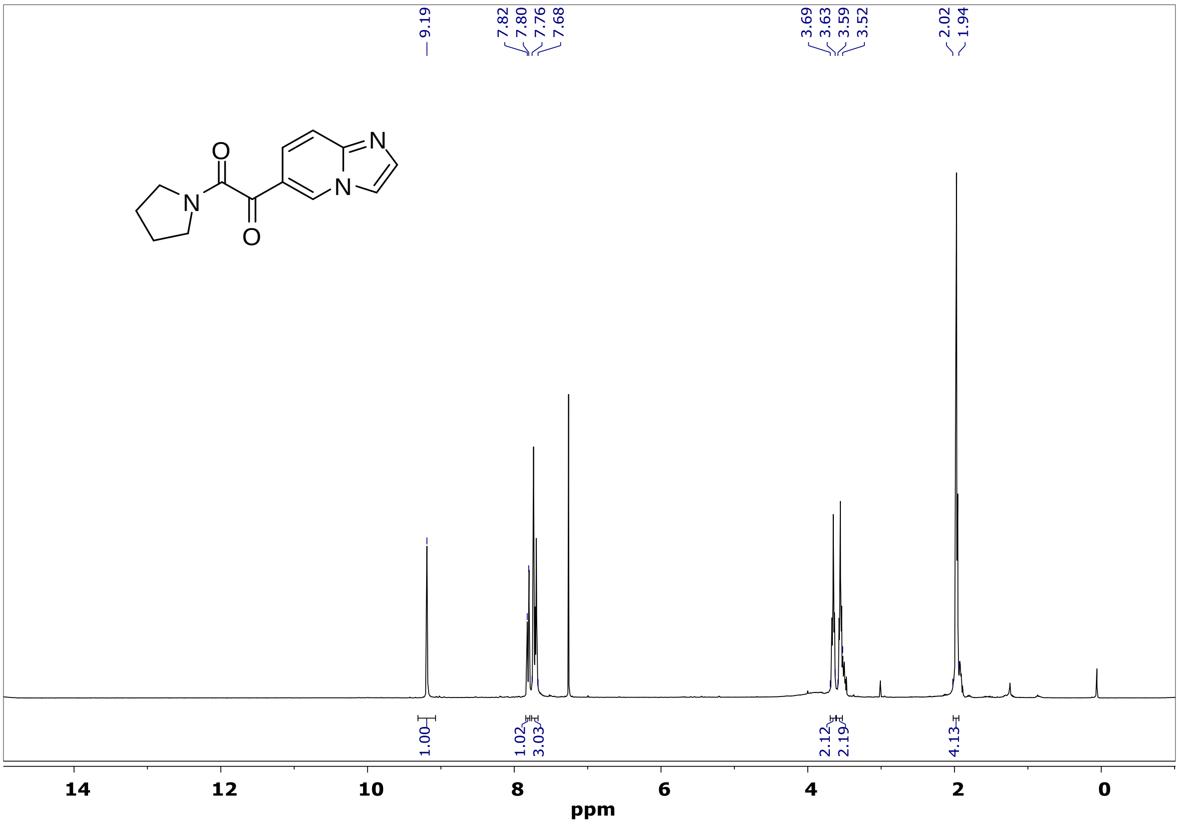

^{1}H NMR spectrum of 1-(imidazo[1,2-*a*]pyridin-6-yl)-2-(pyrrolidin-1-yl)ethane-1,2-dione (**6b**) ($CDCl_3$)

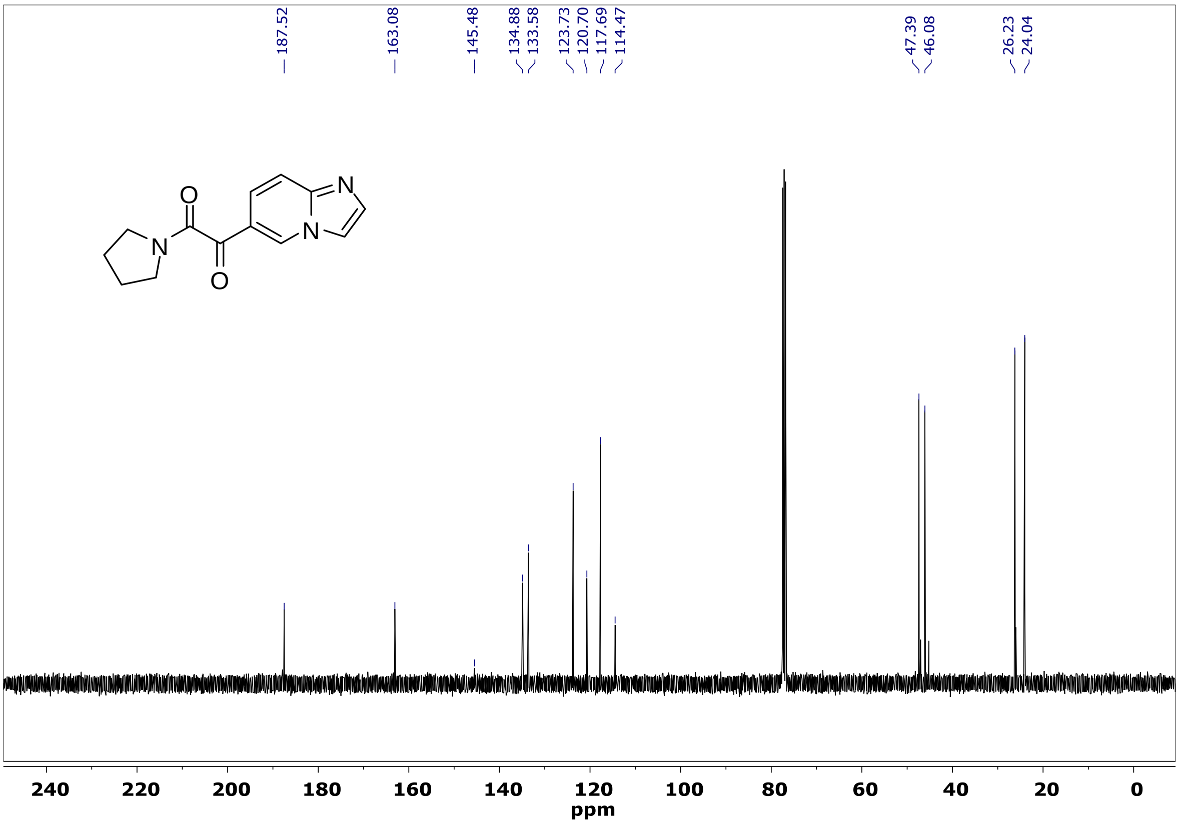

^{13}C{^{1}H} NMR spectrum of 1-(imidazo[1,2-*a*]pyridin-6-yl)-2-(pyrrolidin-1-yl)ethane-1,2-dione (**6b**) ($CDCl_3$)

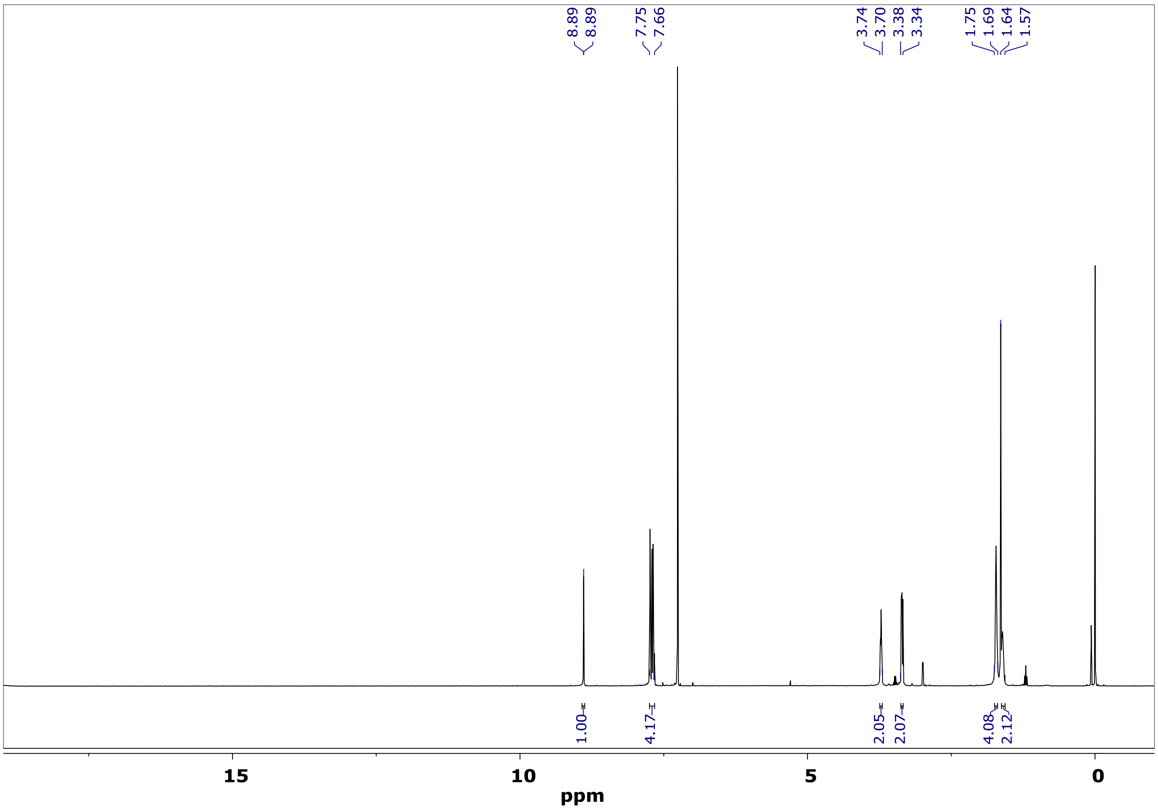

^{1}H NMR spectrum of 1-(imidazo[1,2-*a*]pyridin-6-yl)-2-(piperidin-1-yl)ethane-1,2-dione (**6c**) ($CDCl_3$)

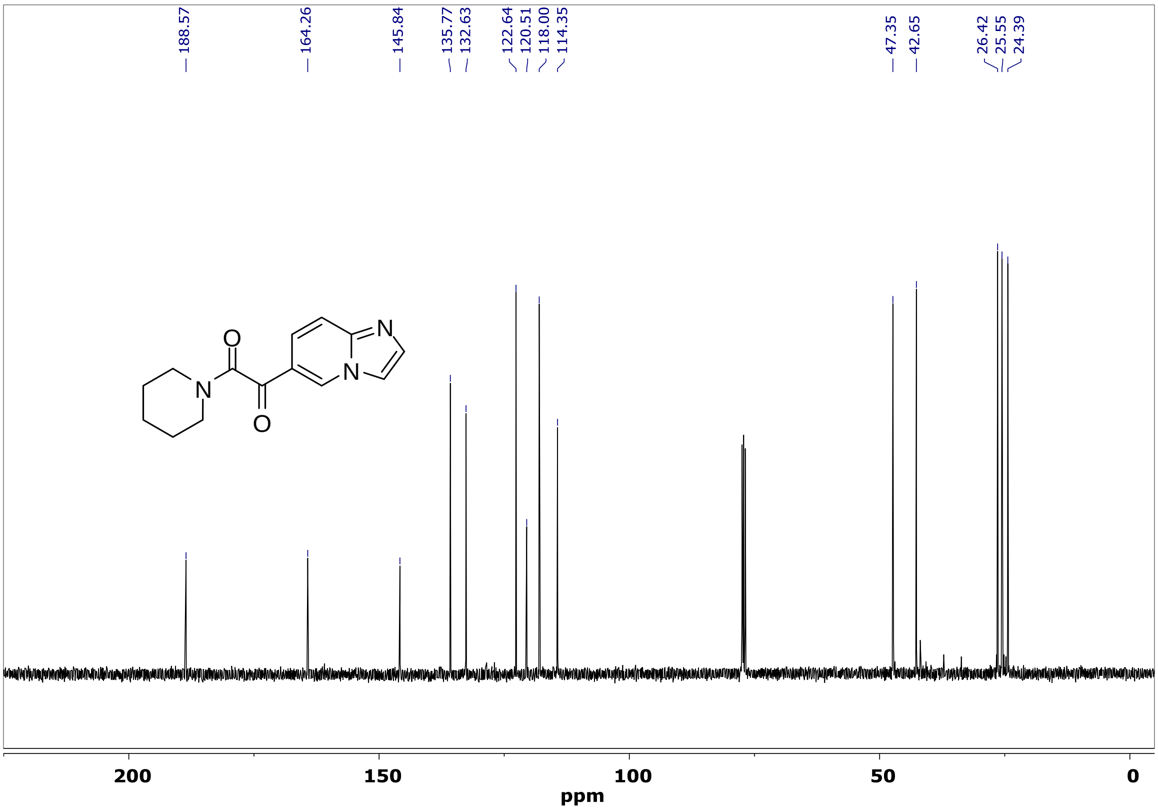

^{13}C{^{1}H} NMR spectrum of 1-(imidazo[1,2-*a*]pyridin-6-yl)-2-(piperidin-1-yl)ethane-1,2-dione (**6c**) ($CDCl_3$)

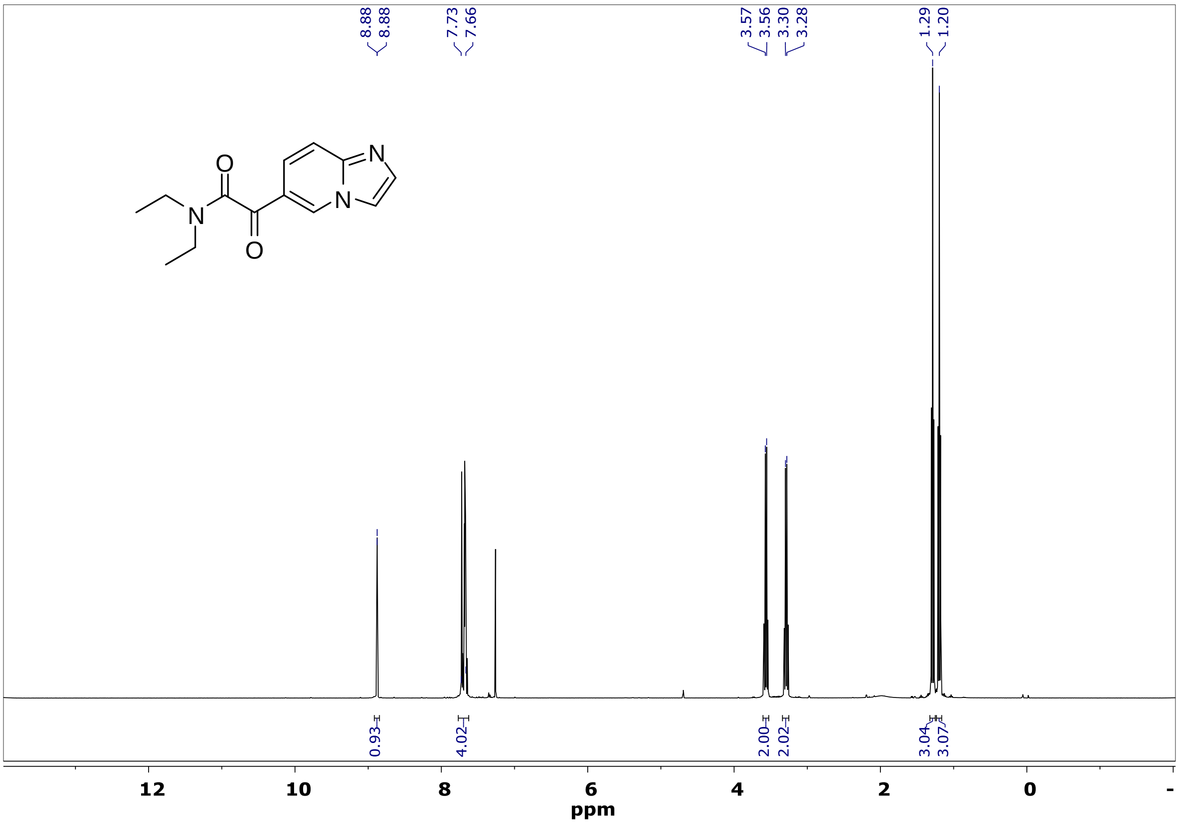

^{1}H NMR spectrum of *N,N*-diethyl-2-(imidazo[1,2-*a*]pyridin-6-yl)-2-oxoacetamide (**6d**) ($CDCl_3$)

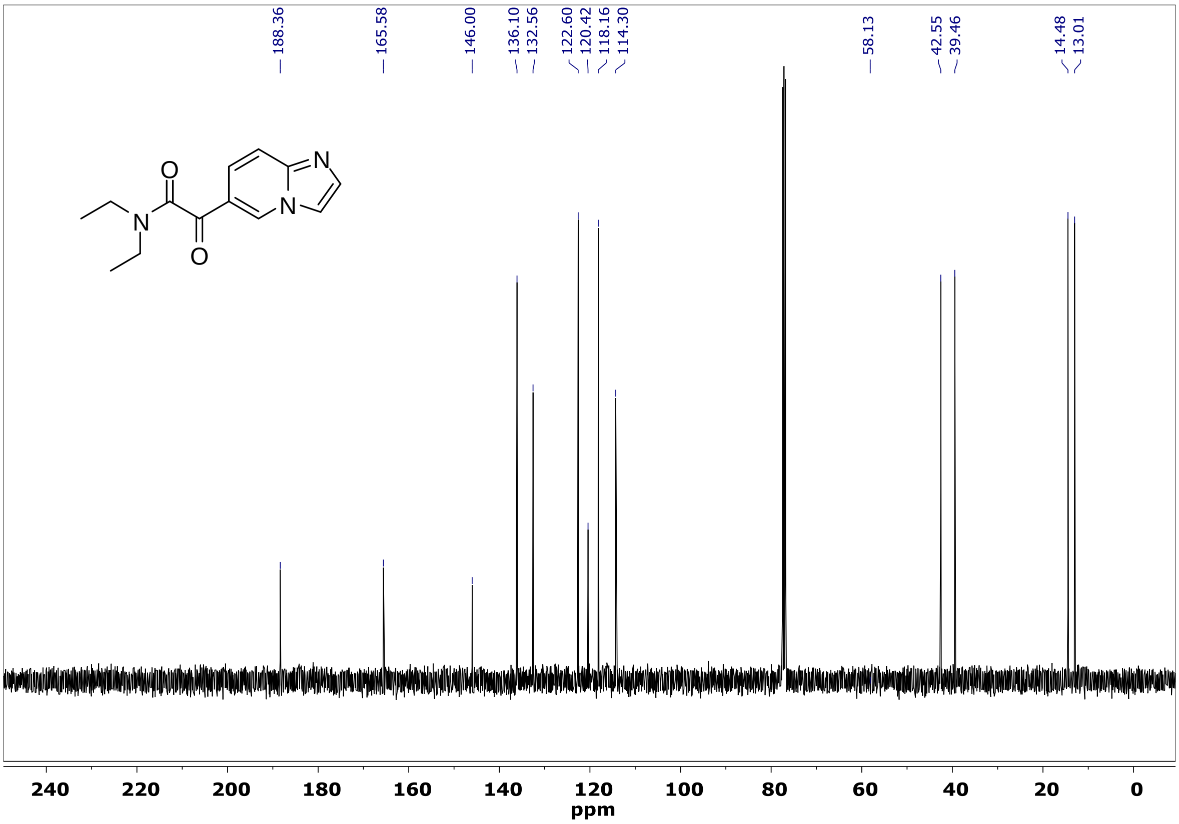

^{13}C{^{1}H} NMR spectrum of *N,N*-diethyl-2-(imidazo[1,2-*a*]pyridin-6-yl)-2-oxoacetamide (**6d**) ($CDCl_3$)

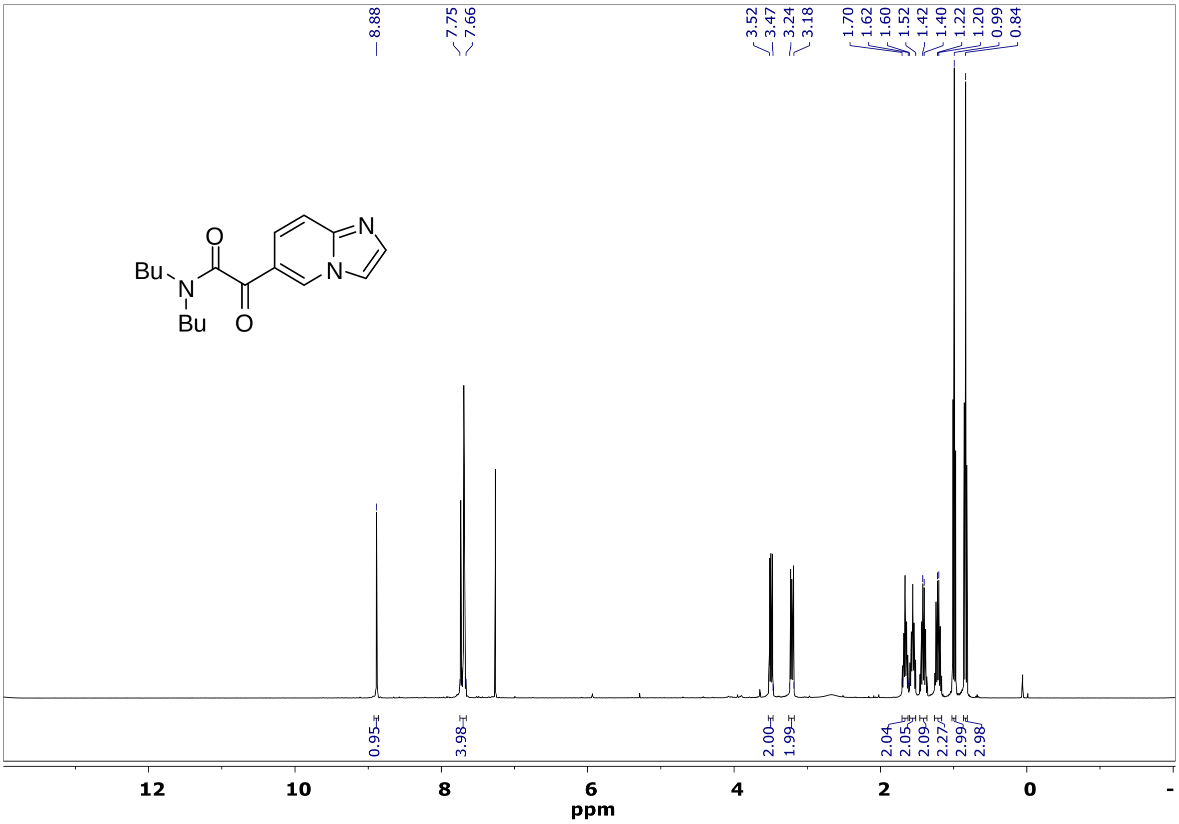

^{1}H NMR spectrum of *N,N*-dibutyl-2-(imidazo[1,2-*a*]pyridin-6-yl)-2-oxoacetamide (**6e**) ($CDCl_3$)

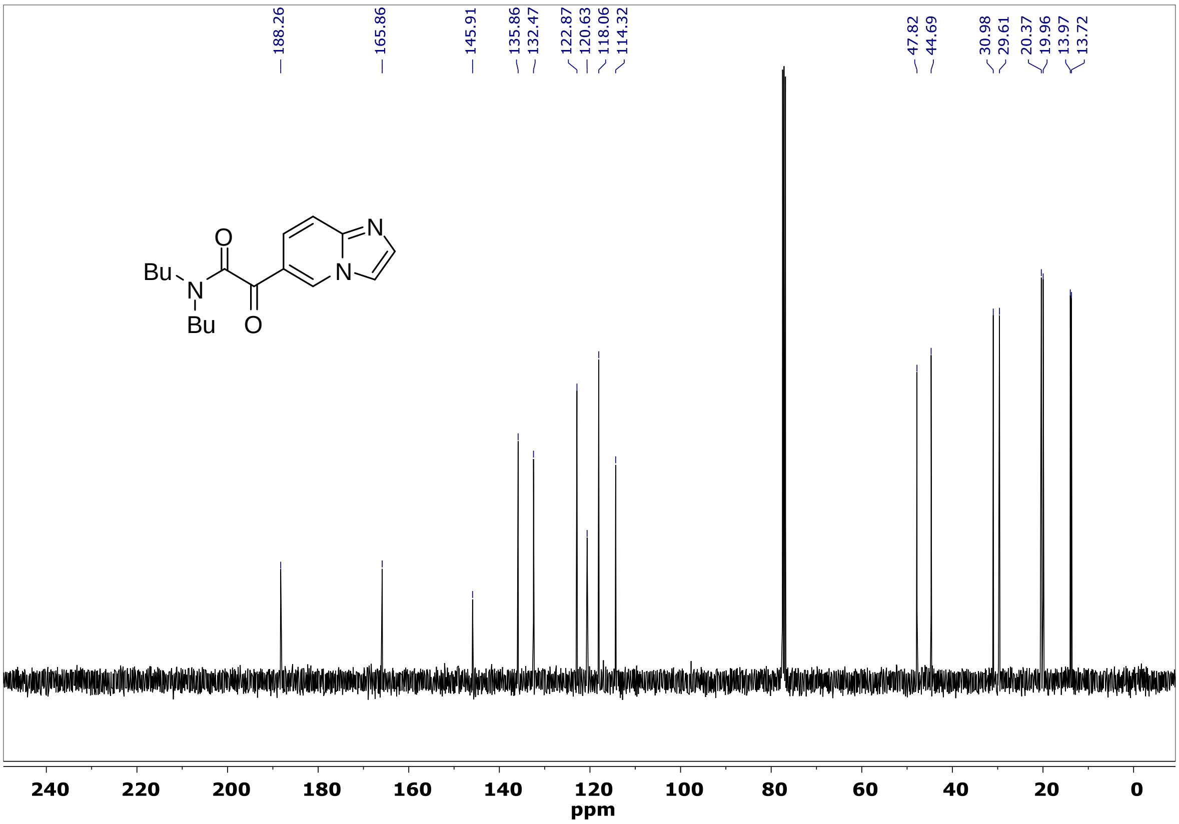

^{13}C{^{1}H} NMR spectrum of *N,N*-dibutyl-2-(imidazo[1,2-*a*]pyridin-6-yl)-2-oxoacetamide (**6e**) ($CDCl_3$)

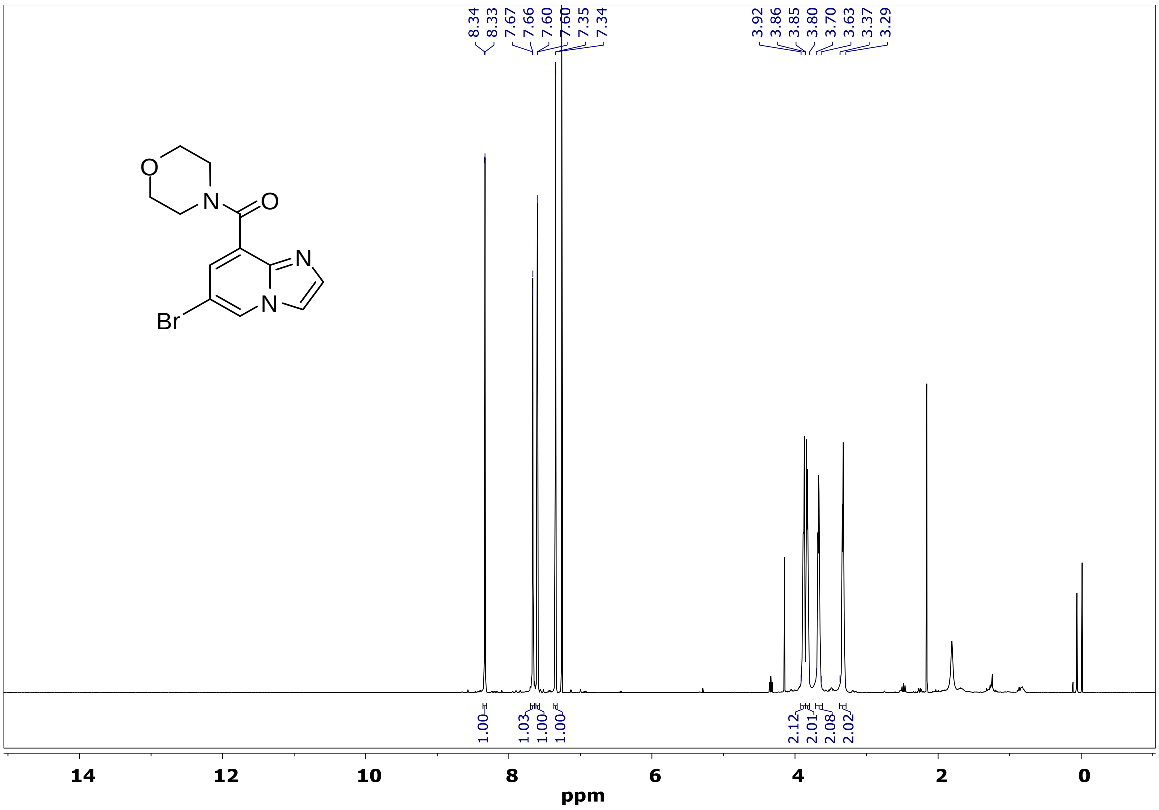

^{1}H NMR spectrum of (6-bromoimidazo[1,2-*a*]pyridin-8-yl)(morpholino)methanone (**9a**) ($CDCl_3$)

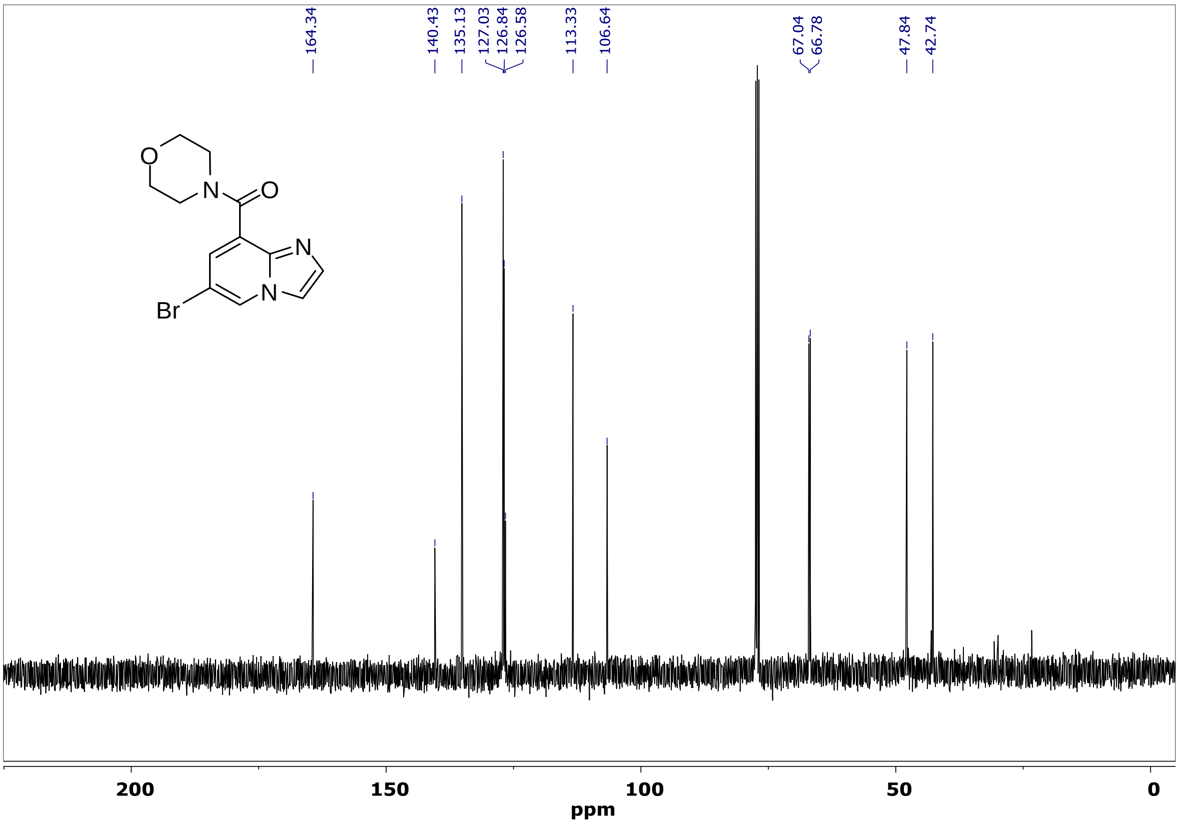

^{13}C{^{1}H} NMR spectrum of (6-bromoimidazo[1,2-*a*]pyridin-8-yl)(morpholino)methanone (**9a**) ($CDCl_3$)

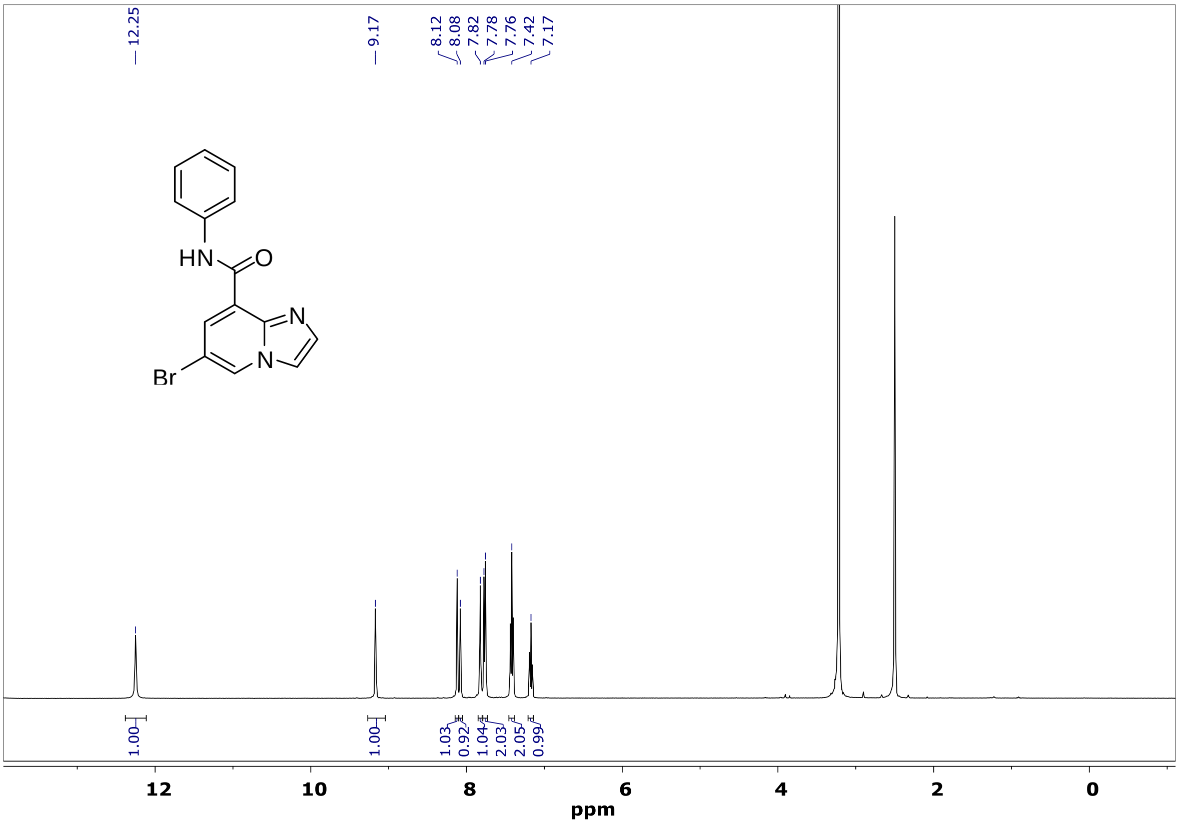

^{1}H NMR spectrum of 6-bromo-*N*-phenyl-imidazo[1,2-*a*]pyridine-8-carboxamide (**9f**) (DMSO-d$_6$)

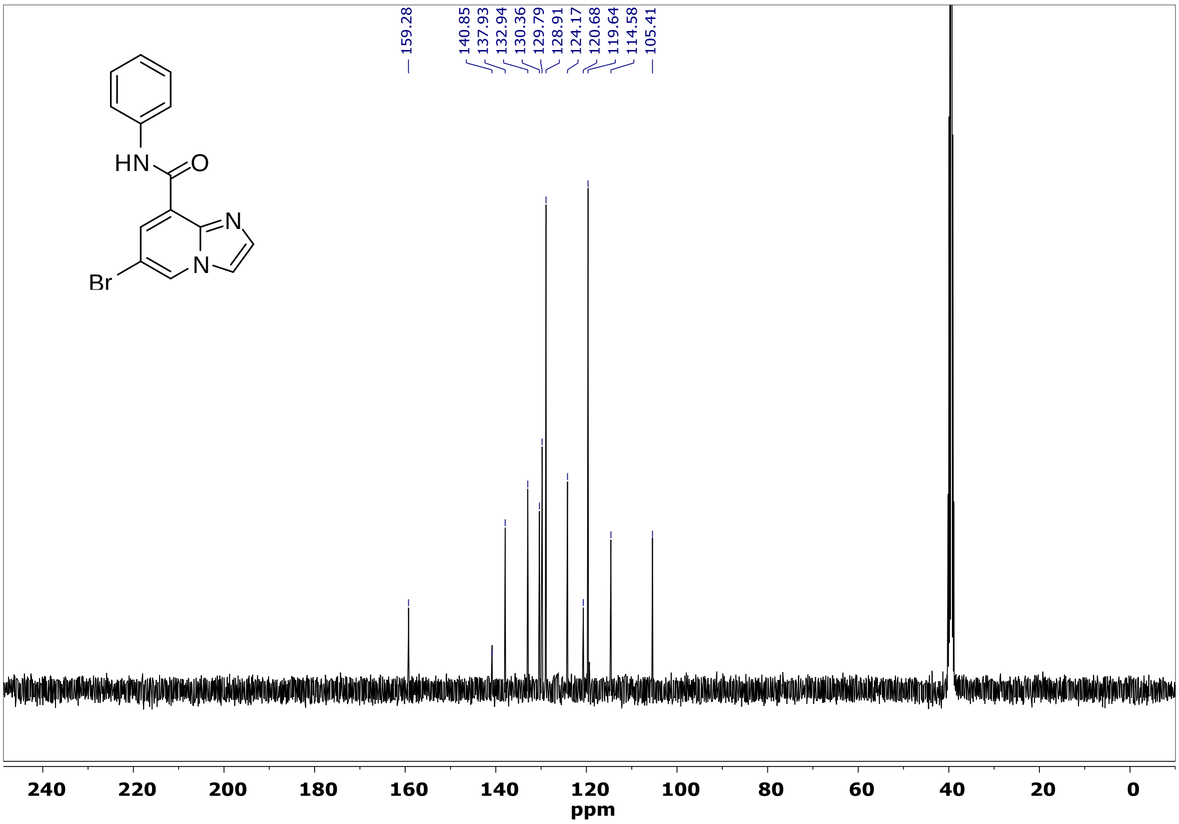

^{13}C{^{1}H} NMR spectrum of 6-bromo-*N*-phenyl-imidazo[1,2-*a*]pyridine-8-carboxamide (**9f**) (DMSO-d$_6$)

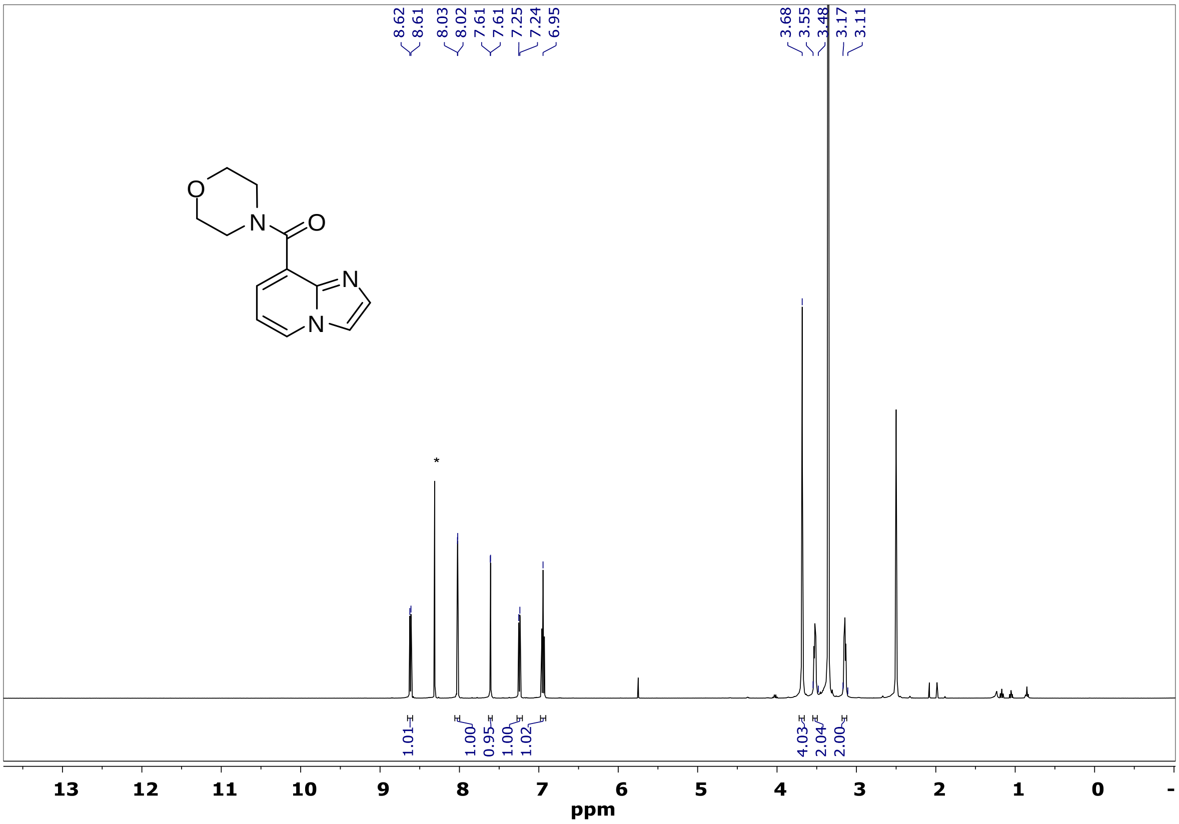

^{1}H NMR spectrum imidazo[1,2-*a*]pyridin-8-yl(morpholino)methanone (**10a**) (DMSO-d6) (*: $CHCl_3$)

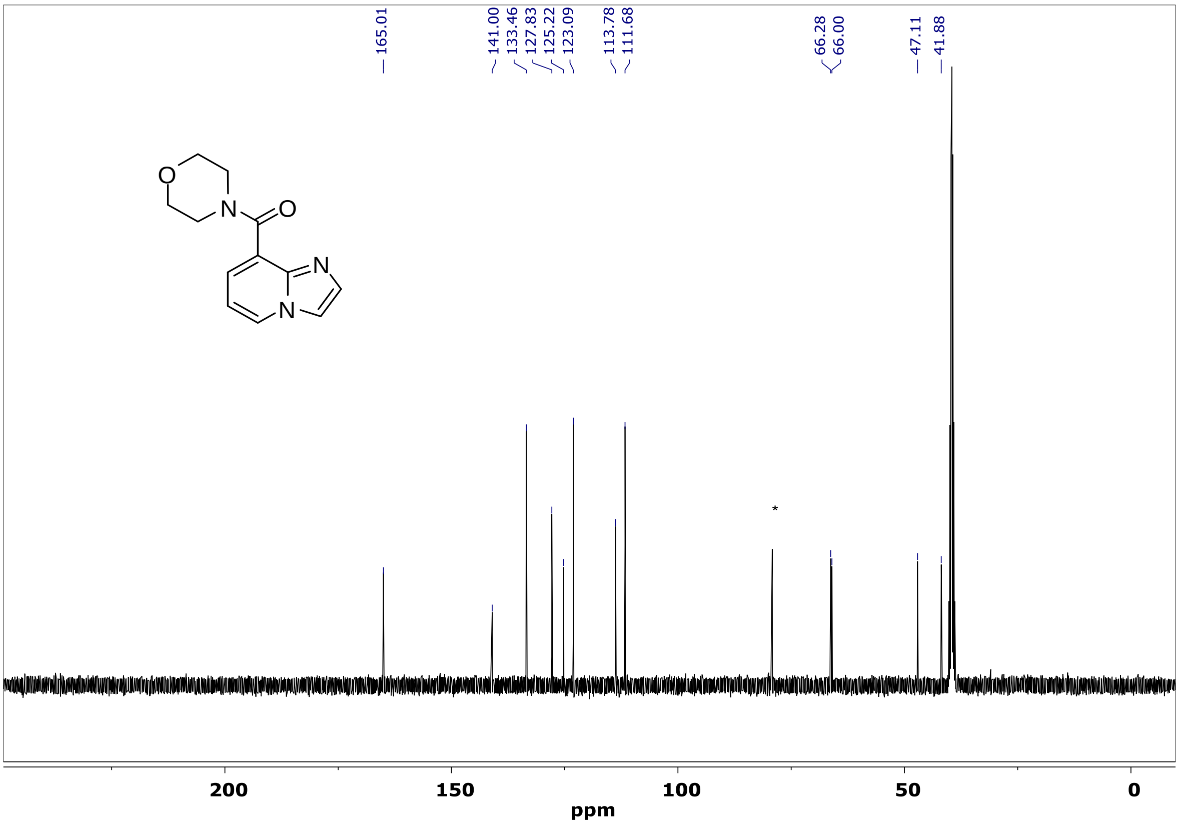

^{13}C{^{1}H} NMR spectrum imidazo[1,2-*a*]pyridin-8-yl(morpholino)methanone (**10a**) (DMSO-d6) (*: $CHCl_3$)
